# Primary-tertiary diamine-catalyzed Michael addition of ketones to isatylidenemalononitrile derivatives

**DOI:** 10.3762/bjoc.10.91

**Published:** 2014-04-24

**Authors:** Akshay Kumar, Swapandeep Singh Chimni

**Affiliations:** 1 Department of Chemistry, U.G.C. Centre of Advance Studies in Chemistry, Guru Nanak Dev University, Amritsar, 143005, India; Fax: (+)91-183-2258820

**Keywords:** 3,3'-disubstituted oxindoles, Michael reaction, organocatalysis, primary-tertiary diamine, spirooxindoles

## Abstract

Simple primary-tertiary diamines easily derived from natural primary amino acids were used to catalyze the Michael addition of ketones with isatylidenemalononitrile derivatives. Diamine **1a** in combination with D-CSA as an additive provided Michael adducts in high yield (up to 94%) and excellent enantioselectivity (up to 99%). The catalyst **1a** was successfully used to catalyze the three-component version of the reaction by a domino Knoevenagel–Michael sequence. The Michael adduct **4a** was transformed into spirooxindole **6** by a reduction with sodium borohydride in a highly enantioselective manner.

## Introduction

**Figure 1:**
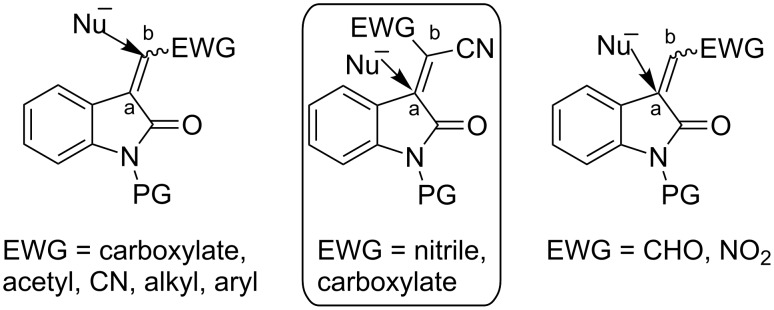
Oxindole based Michael acceptors.

The Michael reaction is one of the fundamental carbon–carbon bond forming reactions in organic synthesis, since a plethora of carbon nucleophiles and activated olefins could be expected to give versatile arrangements [1–8]. Among the various Michael acceptors, the oxindole-based Michael acceptors (Figure 1) are considered as valuable electrophiles for catalytic Michael reactions, as these provide a viable approach to procure 3,3'-disubstituted oxindole frameworks [9–16]. The oxindole framework bearing a tetra-substituted carbon stereocenter at C-3 is a privileged heterocyclic motif that is present in a large variety of bioactive natural products and a series of pharmaceutically active compounds [17]. In recent years, isatins derived Michael acceptors, isatylidenemalononitriles, have attracted considerable attention as novel substrates for the enantioselective synthesis of 3,3'-disubstituted oxindoles [18–22]. The organocatalytic Michael addition of ketones to isatylidenemalononitriles via enamine-catalysis has emerged as a useful tool for the synthesis of 3,3'-disubstituted oxindoles, which serve as important precursors to procure various structurally diverse spirooxindoles [23–24].

**Figure 2:**
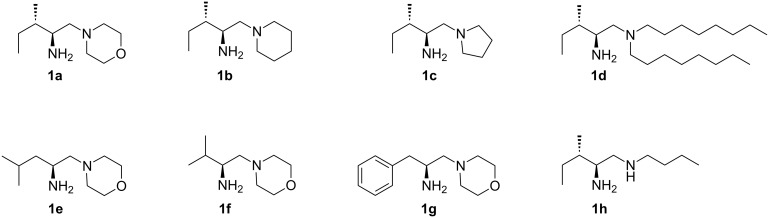
Primary-tertiary diamine organocatalysts.

Over the years, many chiral organocatalysts have been developed and explored for Michael reactions [1–8]. Recently, aminocatalysts – in particular those bearing a primary amine moiety – have been found to catalyze a variety of carbon–carbon bond-forming reactions [25–30]. Small peptides derived from acyclic amino acids, primary-secondary diamines, Cinchona-based primary amines, and thioureas with a primary amine functionality etc., have found many successful applications in Michael addition reactions via an iminium–enamine catalysis [31–37]. A few applications of primary-tertiary diamine in aldol reactions have been published [39–43]. To the best of our knowledge, however, the catalytic potential of amino acids derived primary-tertiary diamine organocatalysts for Michael reaction via enamine activation has not been investigated so far [38]. With readily available and inexpensive natural amino acids as a chiral source, we developed very simple primary-tertiary diamine organocatalysts (Figure 2) for asymmetric aldol reactions [44–45]. We describe herein that a similar catalyst system could also efficiently catalyze the asymmetric Michael addition reaction between ketones **2** and isatylidenemalononitrile derivatives **3** to procure highly functionalized 3,3'-disubstituted oxindoles **4** which could easily be transformed into spirooxindoles.

## Results and Discussion

**Scheme 1:**
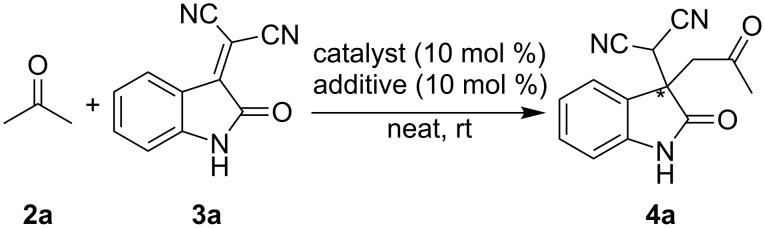
Diamine catalyzed Michael addition of acetone to isatylidenemalononitrile.

Initially, the Michael addition of acetone (**2a**) to isatylidenemalononitrile (**3a**) catalyzed by chiral diamine **1a** (10 mol %) with trichloroacetic acid (10 mol %) as an additive under mild conditions at room temperature was investigated (Scheme 1).

The Michael adduct **4a** was isolated in 95% yield and 69% ee (Table 1, entry 1). Encouraged by the outcome of the preliminary reaction, we optimized the reaction conditions by studying the effect of different solvents and their amount as well as the effect of acid additives on this transformation (Tables 1–3). In our previous studies [10], the diamine **1a** afforded the best result for aldol reactions with water as a solvent. Consequently, the above transformation was performed with water as a solvent. The Michael adduct **4a** was obtained in good yield of 87% and a moderate enantioselectivity of 55% ee (Table 1, entry 2). It was planned to study the effect of different organic solvents on the stereochemical outcome of this reaction. Interestingly, the reaction of **2a** with **3a** performed in tetrahydrofuran (THF) gave Michael adduct **4a** in good yield of 91% and a higher enantioselectivity of 84% ee (Table 1, entry 3). Other etheral solvents, such as 1,4-dioxane and methyl *tert*-butyl ether (MTBE), provided Michael adduct **4a** in 89% and 90% yield and 89% ee and 87% ee, respectively (Table 1, entries 4 and 5). In toluene, **4a** was obtained in 89% yield and 90% ee (Table 1, entry 6). The chlorinated solvents such as dichloromethane, chloroform and 1,2-dichloroethane (DCE) gave **4a** in 90%, 89% and 91% yield and 90% ee, 88% ee and 91% ee, respectively (Table 1, entries 7–9). The utilization of methanol as a solvent led to the isolation of Michael adduct **4a** in good yield but with a low enantioselectivity of 12% ee (Table 1, entry 11). Dimethylformamide was also found to be an inferior solvent for this reaction (Table 1, entry 12). Thus, 1,2-dichloroethane emerged as the solvent of choice for this transformation and was used for all further optimization studies (Table 1, entry 9).

**Table 1:** Solvent screening the Michael addition of acetone (**2a**) to isatylidenemalononitrile (**3a**) catalyzed by **1a** TCA.^a^

Entry	Solvent	Time (h)	Yield (%)^b^	ee (%)^c^

1	–	6	95	69
2	water	48	87	55
3	THF	30	91	84
4	dioxane	30	89	89
5	MTBE	30	90	87
6	toluene	36	89	90
7	DCM	36	90	90
8	CHCl_3_	36	89	88
9	ClCH_2_CH_2_Cl	36	91	91
10	ethyl acetate	36	90	87
11	CH_3_OH	40	88	12
12	DMF	40	82	20

^a^Reaction conditions: 1.5 mmol of acetone (**2a**), 0.125 mmol of isatylidenemalononitrile (**3a**), 0.25 mL of solvent, 10 mol % of catalyst **1a**, 10 mol % of TCA, at 25 °C. ^b^Isolated yield determined after chromatographic purification. ^c^Enantiomeric excess determined by chiral HPLC.

In order to see the effect of the reaction concentration, the amount of 1,2-dichloroethane was varied (Table 2). The use of 0.5 mL of 1,2-dichloroethane afforded **4a** in good yield of 93% and enantioselectivity of 91% ee (Table 2, entry 2). The reaction carried out with 0.75 mL, 1.0 mL and 1.5 mL of 1,2-dichloroethane provided **4a** with an enhanced enantioselectivity of 92% ee, 93% ee and 95% ee, respectively (Table 2, entries 3–5). On performing the reaction in 2.0 mL of 1,2-dichloroethane, **4a** was obtained in 88% yield and increased enantioselectivity of 96% ee after a long reaction time of 96 hours (Table 2, entry 6). There was a small difference in the enantioselectivity of the product **4a** on performing the reaction in 1.5 mL and 2.0 mL of 1,2-dichloroethane, but the rate of the reaction was faster when a lower amount of solvent was used. So, we decided to employ 1.5 mL of 1,2-dichloroethane as a solvent for the further optimization experiments.

**Table 2:** Effect of the amount of solvent (DCE) on the enantioselectivity of the Michael addition of acetone (**2a**) to isatylidenemalononitrile (**3a**) catalyzed by **1a** TCA.^a^

Entry	Amount of solvent (mL)	Time (h)	Yield (%)^b^	ee (%)^c^

1	0.25	24	93	91
2	0.50	26	93	91
3	0.75	32	92	92
4	1.00	40	90	93
5	1.50	60	90	95
6	2.00	96	88	96

^a^Reaction conditions: 1.5 mmol of acetone (**2a**), 0.125 mmol of isatylidenemalononitrile (**3a**), 1,2-dichloroethane (0.25–2.00 mL), 10 mol % of catalyst **1a**, 10 mol % of TCA, at 25 °C. ^b^Isolated yield determined after chromatographic purification. ^c^Enantiomeric excess determined by chiral HPLC.

In order to obtain a highly enantioselective transformation, the effect of different acid additives on the enantioselectivity of **4a** was studied (Table 3). The reaction was performed with 3,5-dinitrobenzoic acid and chloroacetic acid afforded **4a** in good yield of 91% and 90%; and moderate enantioselectivity of 58% ee and 60% ee, respectively (Table 3, entries 1 and 2). The reaction carried out with strong acids such as trifluoromethanesulfonic acid (TsOH) and trifluoroacetic acid (TFA) gave **4a** in good yields of 86% and 89% and high enantioselectivities of 97% ee and 96% ee (Table 3, entries 3 and 4). The application of L-camphorsulfonic acid as an additive resulted in the isolation of **4a** in 92% yield and an enantioselectivity of 98% ee after 30 hours (Table 3, entry 5). The D-camphorsulfonic acid turned out to be the best acid additive providing **4a** in high yield of 93% and excellent enantioselectivity of 99% ee after a reaction time of 24 hours (Table 3, entry 6) [46].

**Table 3:** Additive screening of the **1a** catalyzed Michael addition of acetone (**2a**) to isatylidenemalononitrile (**3a**)^a^.

Entry	Additive	Time (h)	Yield (%)^b^	ee (%)^c^

1	3,5-dinitrobenzoic acid	36	91	58
2	chloroacetic acid	36	90	60
3	TsOH	60	86	97
4	TFA	48	89	96
5	L-CSA	30	92	98
6	D-CSA	24	93	99

^a^Reaction conditions: 1.5 mmol of acetone (**2a**), 0.125 mmol of isatylidenemalononitrile (**3a**), 1.5 mL of DCE, 10 mol % of catalyst **1a**, 10 mol % of additive at 25 °C. ^b^Isolated yield determined after chromatographic purification. ^c^Enantiomeric excess determined by chiral HPLC.

Even though an excellent level of enantioselectivity of the product was observed with catalyst **1a** we screened different diamine catalysts in our quest for a superior catalyst. L-Isoleucine derived primary-tertiary diamine catalysts having piperidinyl **1b** and pyrrolidinyl **1c** groups gave Michael product **4a** in high yield (>90%) and excellent enantioselectivity (98% ee each) (Table 4, entries 2 and 3). The primary-tertiary diamine catalysts characterized by an acyclic tertiary amine, such as a *N*,*N*-dioctyl (**1d**) group, gave **4a** in 89% yield and 96% ee (Table 4, entry 4). The L-leucine, L-valine and L-phenylalanine derived primary-tertiary diamine catalysts (**1e**–**1g**) also provide the Michael adduct **4a** in good yield (92–94%) and an excellent level of enantioselectivity (97–98% ee) (Table 4, entries 5–7). All primary-tertiary diamine catalysts **1a–1g** gave **4a** in high yield (89–94%) with an excellent level of enantioselectivity (96–99% ee). In contrast, the primary-secondary diamine **1h** catalyst afforded Michael adduct **4a** in 10% yield after a long reaction time (Table 4, entry 8). The screening study highlights the importance of the primary-tertiary diamine skeleton in the catalysis of the addition of acetone (**2a**) to isatylidenemalononitrile (**3a**). Thus, the best reaction conditions consist of 10 mol % of catalyst **1a**, 10 mol % of D-camphorsulfonic acid as an additive and 1.5 mL of 1,2-dichloroethane at room temperature providing Michael adduct **4a** in 93% yield and 99% ee.

**Table 4:** Catalyst screening of the enantioselective Michael addition of acetone (**2a**) to isatylidenemalononitrile (**3a**).^a^

Entry	Catalyst	Time (h)	Yield (%)^b^	ee (%)^c^

1	**1a**	24	93	99
2	**1b**	24	93	98
3	**1c**	24	91	98
4	**1d**	30	89	96
5	**1e**	30	93	98
6	**1f**	24	94	98
7	**1g**	30	92	97
8	**1h**	96	10	–

^a^Reaction conditions: 1.5 mmol of acetone (**2a**), 0.125 mmol of isatylidenemalononitrile (**3a**), 1.5 mL of 1,2-dichloroethane, 10 mol % of catalyst **1a**–**1h**, 10 mol % of D-CSA at 25 °C. ^b^Isolated yield determined after chromatographic purification. ^c^Enantiomeric excess determined by chiral HPLC.

**Scheme 2:**
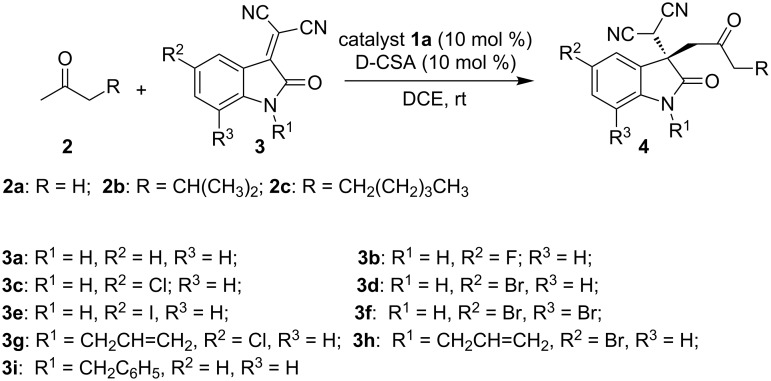
Substrate scope of the addition of **2** with **3** catalyzed by **1a** D-CSA.

Once the optimized reaction conditions have been found, the substrate scope was investigated by using different ketones **2a–c** and isatylidenemalononitrile derivatives **3a–i** (Scheme 2). The methodology was found to be suitable for both N-substituted isatylidenemalononitrile and *N*–H isatylidenemalononitrile derivatives. Acetone (**2a**) reacts well with various *N*–H isatylidenemalononitrile derivatives (**3a–f**) providing corresponding Michael adducts **4a–f** in excellent enantioselectivity (96–99% ee) after a reaction time of 24–36 hours. The 5-fluoroisatylidenemalononitrile (**3b**), 5-chloroisatylidenemalononitrile (**3c**), 5-bromoisatylidenemalononitrile (**3d**) and 5-iodoisatylidenemalononitrile (**3e**) gave corresponding Michael adducts **4b–e** in 93%, 95%, 94% and 91% yield and 98% ee, 98% ee, 98% ee and 99% ee, respectively (Table 5, entries 2–5). The reaction of 5,7-dibromoisatylidenemalononitrile (**3f**) with acetone (**2a**) proceeds with a high yield of 92% and a high enantioselectivity of 96% ee (Table 5, entry 6). The N-substituted isatylidenemalononitriles **3g–i** react slowly with acetone (**2a**) to afford Michael adducts **4g–i** in good yield (85–87%) and good enantioselectivity (88–92% ee) (Table 5, entries 7–9). Acetone (**2a**) reacts well with *N*-allyl isatylidenemalononitrile derivatives **3g** and **3h** to provide the respective Michael adducts **4g** and **4h** in 85% and 87% yield and 89% ee and 92% ee, respectively (Table 5, entries 7 and 8). Using *N*-benzyl isatylidenemalononitrile (**3i**), the Michael adduct **4i** was isolated in 86% yield and 88% ee (Table 5, entry 9). A recently reported similar reaction catalyzed by Cinchona alkaloid-based primary amine catalyst requires high catalyst loading and is only suitable for N-unprotected isatylidenemalononitrile derivatives [5]. In contrast, our methodology is suitable for both N-unprotected and N-protected isatylidenemalononitrile derivatives and is highly enantioselective. Next, the substrate scope of the reaction was extended to different acyclic ketones **2b** and **2c**. Under the optimized conditions, the Michael addition of methyl isobutyl ketone (**2b**) and 2-octanone (**2c**) with **3a** provided Michael adducts **4j** and **4k** in 24% and 41% yield and 97% ee and 96% ee, respectively (Table 5, entries 10 and 11). Due to the low reactivity of these ketones, these experiments were carried out after a slight modification of the optimized conditions, i.e., a higher catalyst loading of 20 mol % of **1a** and 1.0 mL of 1,2-dichloroethane as a solvent. The 20 mol % of **1a** catalyzes the Michael addition of **2b** with **3a** providing Michael adduct **4j** in 80% yield and 96% ee after a reaction time of 7 days (Table 5, entry 12). The 2-octanone (**2c**) gave Michael adduct **4k** in 85% yield and 97% ee after a reaction time of 6 days (Table 5, entry 13). The *R* absolute configuration of Michael adducts was assigned by comparing the HPLC chromatograms of Michael adducts with that reported in the literature [23–24].

**Table 5:** Substrate scope of **1a** D-CSA catalyzed asymmetric Michael reaction of ketones **2** with isatylidenemalononitrile derivatives **3**.^a^

Entry	**2**	**3**	Time (h)	**4**	Yield (%)^b^	ee (%)^c^

1	**2a**	**3a**	26	**4a**	92	99
2	**2a**	**3b**	26	**4b**	93	98
3	**2a**	**3c**	26	**4c**	95	98
4	**2a**	**3d**	30	**4d**	94	98
5	**2a**	**3e**	36	**4e**	91	99
6	**2a**	**3f**	24	**4f**	92	96
7	**2a**	**3g**	72	**4g**	85	89
8	**2a**	**3h**	78	**4h**	87	92
9	**2a**	**3i**	78	**4i**	86	88
10	**2b**	**3a**	168	**4j**	24	97
11	**2c**	**3a**	168	**4k**	41	96
12^d^	**2b**	**3a**	168	**4j**	80	96
13^d^	**2c**	**3a**	144	**4k**	85	97

^a^Reaction conditions: 1.5 mmol of ketones **2**, 0.125 mmol of isatylidenemalononitrile derivatives **3**, 1.5 mL of 1,2-dichloroethane, 10 mol % of catalyst **1a**, 10 mol % of additive D-CSA at 25 °C. ^b^Isolated yield determined after chromatographic purification. ^c^Enantiomeric excess determined by chiral HPLC. ^d^The reaction is performed with 20 mol % catalyst **1a** and 1.0 mL of 1,2-dichloroethane.

**Scheme 3:**
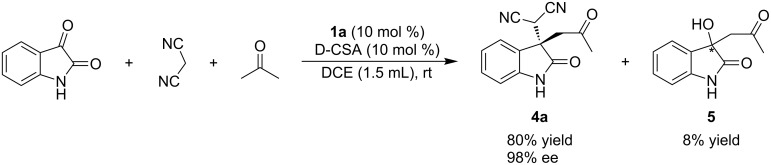
One-pot, three-component Knoevenagel condensation–Michael addition.

Next, we studied the multicomponent version of this reaction. Acetone, isatin and malononitrile react in one pot providing Michael product **4a** in a good yield of 80% and a high enantioselectivity of 98% ee (Scheme 3). The slightly lower yield of the one-pot process compared to the stepwise process was due to the competing reaction of isatin and acetone to form aldol adduct **5** (8% yield). The reaction involves the initial formation of isatylidenemalononitrile by Knoevenagel condensation of isatin with malononitrile followed by the addition of acetone to provide Michael adduct **4a**. Thus, catalyst **1a** also finds its successful application in the multicomponet version of this reaction without compromising enantioselectivity – albeit with a slight loss in yield.

**Scheme 4:**
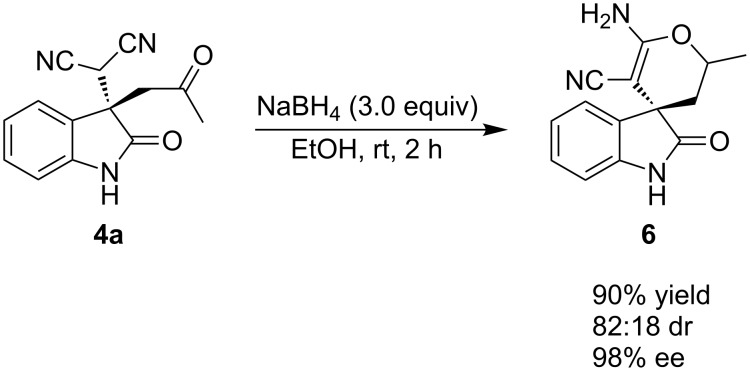
Cascade reduction–cyclization for the synthesis of spirooxindole.

We further demonstrated that Michael adducts could be transformed into spirooxindoles by following a simple strategy. The reduction of Michael adduct **4a** with sodium borohydride in ethanol followed by spontaneous cyclization gave spirooxindole product **6** in 90% yield, 82:18 dr and 98% ee (Scheme 4).

## Conclusion

In conclusion, we successfully demonstrated the use of the very simple primary-tertiary diamine catalyst **1a** in combination with D-CSA as an additive for the enantioselective catalysis of a Michael addition of acetone to isatylidenemalononitriles. A three component process involving a domino Knoevenagel–Michael sequence was developed. 3,3'-Disubstituted oxindole could be transformed into spirooxindoles by reduction with NaBH_4_.

## Supporting Information

File 1 Experimental procedures, copies of ^1^H and ^13^C NMR spectra of Michael adducts, and HPLC chromatogram of products **4**.
