# Supplementary material for: Primary-tertiary diamine-catalyzed Michael addition of ketones to isatylidenemalononitrile derivatives
Source: Beilstein J Org Chem. 2014 Apr 24;10:929–35. doi: 10.3762/bjoc.10.91 (PMC4077384; doi:10.3762/bjoc.10.91)
Supplement: File 1 — Experimental procedures, copies of 1H and 13C NMR spectra of Michael adducts, and HPLC chromatogram of products 4. [file Beilstein_J_Org_Chem-10-929-s001.pdf]

Supporting Information

for

Primary-tertiary diamine-catalyzed Michael addition of ketones to isatylidenemalononitrile derivatives

Akshay Kumar and Swapandeep Singh Chimni*

Address: Department of Chemistry, U.G.C. Centre of Advance Studies in Chemistry, Guru Nanak Dev University, Amritsar, 143005, India; Fax: (+)91-183-2258820

Email: Swapandeep Singh Chimni - sschimni@yahoo.com

*Corresponding author

Experimental procedures, copies of ^{1}H and ^{13}C NMR spectra of Michael adducts and HPLC chromatogram of products **4**

Table of Contents

1. General remarks..S2

2. Experimental procedures S2

3. Experiment data S4

4. ^{1}H and ^{13}C spectra of Michael adducts **4**...S10

5. HPLC chromatograms of Michael adducts **4** S21

General Remarks

All reagents were commercially available and were used without purification. Isatylidenemalononitrile derivatives **3** were prepared according to the reported procedure [1]. The primary-tertiary diamines were prepared according to our previously reported method [2]. NMR spectra were obtained at 300 MHz for ^{1}H NMR and at 75 MHz for ^{13}C NMR in $CDCl_3$ with Me_4Si as internal standard. The chemical shifts are reported on δ scale relative to TMS and coupling constants *J* are expressed in Hz. Spectral patterns are designated as s = singlet; d = doublet; dd = doublet of doublet; t = triplet; br = broad; m = multiplet. Optical rotations were determined on a digital polarimeter. Column chromatography was carried out on a with silica gel (60–120 mesh) using increasing concentration of ethyl acetate in hexane as eluent. HPLC analysis was performed using Daicel Chiralpak AD-H, AS-H, OD-H and IB columns.

General procedure for enantioselective Michael addition of ketone 2 to isatylidenemalononitriles 3.

To a stirred mixture of catalyst **1a** (2.33 mg, 0.0125 mmol), ketone **2** (0.11 mL, 1.5 mmol) in 1,2-dichloroethane (1.5 mL), the additive D-CSA (2.91 mg, 0.0125 mmol) was added at 25 °C and the mixture was allowed to stir for 2 min followed by the addition of the corresponding isatylidenemalononitrile derivative (0.125 mmol). The resulting mixture was stirred for 24–168 h and the progress of the reaction was monitored at regular intervals by TLC. After completion of the reaction, saturated solution of NH_4Cl (3 mL) was added and the resulting mixture was extracted with ethyl acetate (3 × 10 mL). The organic layer was separated, dried over anhydrous sodium sulfate, filtered, and concentrated under reduced pressure to

obtain the crude Michael product. Subsequent purification by column chromatography gave the corresponding pure Michael adducts. The enantiomeric excess of the products was determined using Diacel Chiralpak AS-H, OD-H, AD-H and IB columns. Racemic standards were prepared using (±)-3-methyl-1-morpholinobutan-2-amine as catalyst synthesized from (±)-valine.

General Procedure for three component enantioselective Michael reaction

To a stirred mixture of catalyst **1a** (2.33 mg, 0.0125 mmol), acetone (0.110 mL, 1.5 mmol), malononitrile (0.137 mmol) in DCE (1.5 mL), the additive D-CSA (2.91 mg, 0.0125 mmol) and isatin (0.125 mmol) was added at 25 °C. The reaction mixture was stirred for 24 h and quenched with saturated solution of NH_4Cl (3 mL). After extraction with ethyl acetate (3 × 10 mL) the organic layer was separated, dried over anhydrous sodium sulfate, filtered, and concentrated under reduced pressure to give the crude Michael product. Purification by column chromatography on silica gel using hexane/ethyl acetate 7:3 as eluent gave the corresponding Michael adduct in 80% yield. The enantiomeric excess of the products was determined using Diacel Chiralpak AD-H.

Procedure for synthesis of spirooxindole [3].

To a stirred solution of **4a** (63 mg, 0.25 mmol) in ethanol (2.5 mL) $NaBH_4$ (28.5 mg, 0.75 mmol) was added. The reaction mixture was stirred for 2 h at rt and quenched with saturated solution of NH_4Cl (5 mL). After extraction with ethyl acetate the organic layer was separated, dried over anhydrous sodium sulfate, filtered, and concentrated under reduced pressure to obtain the crude product **6**. Purfication by column chromatography on silica gel using hexane/ethyl acetate 6:4 as eluent gave spirooxindole product **6** in 90% yield. The diastereomeric ratio was determined by

^{1}H NMR analysis of the pure product. In order to determine the enantiomeric excess of **6**, a racemic sample of spirooxiindole **6** was prepared from racemic Michael adduct **4a** following the same procedure. The enantiomeric excess of spiroxindole **6** was determined using Diacel Chiralpak AD-H column and found to be 98% ee.

2-[(*S*)-2'-Oxo-3'-(2''-oxoprop-1''-yl)indolin-3'-yl]malononitrile (4a)

Sticky solid; yield = 92%; $[\alpha]_D^{25}$ = +24 (c 0.1, MeOH); ee = 99% determine by HPLC [Diacel chiralpak AD-H, hexane/*i*-PrOH, 80:20, 1.0 mL/min, λ = 254 nm, t_R (minor) = 10.2 min, t_R (major) = 13.8 min)]; ^{1}H NMR (300 MHz, DMSO-d_6): *δ* 2.03 (s, 3H, CH_3), 3.27 (d, J = 18.3 Hz, 1H, CH_2), 3.60 (d, J = 18.3 Hz, 1H, CH_2), 5.53 (s, 1H, CH), 6.92 (d, J = 8.1 Hz, 1H, ArH), 6.98-7.03 (m, 1H, ArH), 7.26-7.37 (m, 2H, ArH), 11.00 (s, 1H, NH); ^{13}C NMR (75 MHz, DMSO-d_6): *δ* 29.5, 29.6, 45.4, 48.4, 109.8, 111.3, 111.6, 123.8, 125.8, 128.6, 129.8, 142.1, 175.1, 203.6; m/z (ESI-TOF): 276.0796 (M^++Na).

2-[(*S*)-5'-Fluoro-2'-oxo-3'-(2''-oxoprop-1''-yl)indolin-3'-yl]malononitrile (4b)

Sticky solid; yield = 93%; $[\alpha]_D^{25}$ = +28 (c 0.1, MeOH); ee = 98% determined by HPLC [Diacel Chiralpak AS-H; hexane/*i*-PrOH 80 : 20; flow rate 1 mL/min; λ = 254 nm; t_R (major) = 19.1 min, t_R (major) = 50.4 min]; ^{1}H NMR (300 MHz, DMSO-d_6): *δ* 2.05 (s, 3H, CH_3), 3.30 (d, J = 18.3 Hz, 1H, CH_2), 3.65 (d, J = 18.3 Hz, 1H, CH_2), 5.54 (s, 1H, CH), 6.90-6.95 (m, 1H, ArH), 7.11-7.16 (m, 1H, ArH), 7.27 (d, J = 6.3 Hz, 1H, ArH); ^{13}C NMR (75 MHz, DMSO-d_6): *δ* 29.7, 29.9, 45.6, 48.6, 111.0, 111.3, 111.7, 116.1, 116.4, 128.2, 139.4, 156.2, 159.3, 175.3, 203.6; m/z (ESI-TOF): 294.0649 (M^++Na)

2-[(*S*)-5'-Chloro-2'-oxo-3'-(2''-oxoprop-1''-yl)indolin-3'-yl]malononitrile (4c)

Sticky solid; yield = 95%; $[\alpha]_D^{25}$ = +32 (c 0.1, MeOH); ee = 98% determined by HPLC [Diacel Chiralpak AS-H; hexane/*i*-PrOH 80 : 20; flow rate 1 mL/min; λ = 254 nm; t_R (major) = 20.2 min, t_R (major) = 45.1 min]; ^{1}H NMR (300 MHz, DMSO-d_6): *δ* 2.05 (s, 3H, CH_3), 3.31 (d, J = 18.3 Hz, 1H, CH_2), 3.68 (d, J = 18.3 Hz, 1H, CH_2), 5.55 (s, 1H, CH), 6.94 (d, J = 8.1 Hz, 1H, ArH), 7.33-7.45 (m, 2H, ArH), 11.12 (s, 1H, NH); ^{13}C NMR (75 MHz, DMSO-d_6): *δ* 29.6, 29.8, 45.6, 48.4, 111.0, 111.3, 111.5, 123.8, 125.8, 128.6, 129.8, 142.1, 175.1, 203.6; m/z (ESI-TOF): 310.0349 (M^++Na).

2-[(*S*)-5'-Bromo-2'-oxo-3'-(2''-oxoprop-1''-yl)indolin-3'-yl]malononitrile (4d)

Sticky solid; yield = 94%; $[\alpha]_D^{25}$ = +28 (c 0.1, MeOH); ee = 98% determined by HPLC [Diacel Chiralpak AS-H; hexane/*i*-PrOH 70 : 30; flow rate 1 mL/min; λ = 254 nm; t_R (major) = 11.5 min, t_R (minor) = 23.5 min]; ^{1}H NMR (300 MHz, DMSO-d_6): *δ* 2.05 (s, 3H, CH_3), 3.32 (d, J = 18.0 Hz, 1H, CH_2), 3.68 (d, J = 18.0 Hz, 1H, CH_2), 5.55 (s, 1H, CH), 6.89 (d, J = 8.4 Hz, 1H, ArH), 7.46-7.57 (m, 2H, ArH), 11.13 (s, 1H, NH); ^{13}C NMR (75 MHz, DMSO-d_6): *δ* 29.6, 29.8, 45.6, 48.3, 110.9, 111.3, 112.0, 113.4, 126.5, 128.9, 132.6, 142.5, 175.0, 203.7; m/z (ESI-TOF): 353.9842 (M^++Na).

2-[(*S*)-5'-Iodo-2'-oxo-3'-(2''-oxoprop-1''-yl)indolin-3'-yl]malononitrile (4e)

Sticky solid; yield = 91%; $[\alpha]_D^{25}$ = +21 (c 0.1, MeOH); ee = 99% determined by HPLC [Diacel Chiralpak AS-H; hexane/*i*-PrOH 70 : 30; flow rate 1 mL/min; λ = 254 nm; t_R (major) = 12.1 min, t_R (minor) = 23.5 min]; ^{1}H NMR (300 MHz, DMSO-d_6): δ 2.05 (s, 3H, CH_3), 3.29 (d, J = 18.3 Hz, 1H, CH_2), 3.67 (d, J = 18.3 Hz, 1H, CH_2), 5.54 (s, 1H, CH), 6.78 (d, J = 8.1 Hz, 1H, ArH), 7.62-7.69 (m, 2H, ArH); 11.25 (s, 1H, NH); ^{13}C NMR (75 MHz, DMSO-d_6): *δ* 29.6, 29.8, 45.6, 48.1, 84.6, 111.0, 111.3, 112.4, 129.2, 131.8, 138.4, 142.9, 174.8, 203.7; m/z (ESI-TOF): 401.9715 (M^++Na).

2-[(*S*)-5',7'-Dibromo-2'-oxo-3'-(2''-oxoprop-1''-yl)indolin-3'-yl]malononitrile (4f)

Sticky solid; yield = 92%; $[\alpha]_D^{25}$ = +51 (c 0.1, MeOH); ee = 96% determined by HPLC [Diacel Chiralpak AD-H; hexane/*i*-PrOH 90 : 10; flow rate 1 mL/min; λ = 254 nm; t_R (minor) = 22.4 min, t_R (major) = 30.9 min]; ^{1}H NMR (300 MHz, DMSO-d_6): *δ* 2.06 (s, 3H, CH_3), 3.37 (d, J = 18.3 Hz, 1H, CH_2), 3.73 (d, J = 18.3 Hz, 1H, CH_2), 5.60 (s, 1H, CH), 7.60 (s, 1H, ArH), 7.77 (s, 1H, ArH), 11.49 (s, 1H, NH); ^{13}C NMR (75 MHz; DMSO-d_6): *δ* 29.5, 29.8, 45.8, 49.2, 103.1, 110.7, 111.0, 113.9, 125.7, 130.0, 134.6, 142.2, 174.8, 203.8; m/z (ESI-TOF): 431.9015 (M^++Na).

2-[(*S*)-1'-Allyl-5'-chloro-2'-oxo-3'-(2''-oxoprop-1''-yl)indolin-3'-yl]malononitrile (4g)

Sticky solid; yield = 85%; $[\alpha]_D^{25}$ = +38 (c 0.1, MeOH); ee = 89% determined by HPLC [Diacel Chiralpak AS-H; hexane/*i*-PrOH 80 : 20; flow rate 1 mL/min; λ = 254 nm; t_R (major) = 10.7 min, t_R (minor) = 25.4 min]; ^{1}H NMR (300 MHz, $CDCl_3$): *δ* 2.08 (s, 3H, CH_3), 3.12 (d, J = 18.0 Hz, 1H, CH_2), 3.42 (d, J = 18.0 Hz, 1H, CH_2), 4.35-4.437 (m, 3H, NCH_2 and CH), 5.22-5.38 (m, 2H, CH=CH_2), 5.74-5.84 (m, 1H, C*H*=CH_2), 6.84 (d, J = 8.4 Hz, 1H, ArH), 7.29-7.37 (m, 2H, ArH); ^{13}C NMR (75 MHz, $CDCl_3$): *δ* 30.7, 30.9, 44.7, 47.3, 49.6, 109.7, 110.8, 112.1, 124.5, 127.2, 129.7, 131.4, 142.9, 173.6, 202.9; m/z (ESI-TOF): 350.0654 (M^++Na).

2-[(*S*)-1'-Allyl-5'-bromo-2'-oxo-3'-(2''-oxoprop-1''-yl)indolin-3'-yl]malononitrile (4h)

Sticky solid; yield = 87%; $[\alpha]_D^{25}$ = +35 (c 0.1, MeOH); ee = 92% determined by HPLC [Diacel Chiralpak AS-H; hexane/*i*-PrOH 80 : 20; flow rate 1 mL/min; λ = 254 nm; t_R (major) = 12.8 min, t_R (minor) = 37.9 min]; ^{1}H NMR (300 MHz, $CDCl_3$): *δ* 2.14 (s, 3H, CH_3), 3.20 (d, J = 18.3 Hz, 1H, CH_2), 3.49 (d, J = 18.3 Hz, 1H, CH_2), 4.40-4.42

(m, 2H, NCH_2), 4.46 (s, 1H, CH), 5.28-5.45 (m, 2H, $CH=CH_2$), 5.80-5.90 (m, 1H, $CH=CH_2$), 6.86 (d, J = 8.4 Hz, 1H, ArH), 7.51-7.57 (m, 2H, ArH); ^{13}C NMR (75 MHz, $CDCl_3$): δ 29.9, 30.1, 43.2, 46.6, 48.8, 109.1, 110.1, 111.8, 116.1, 118.7, 126.4, 126.9, 130.0, 133.6, 142.7, 172.8, 202.3; m/z (ESI-TOF): 396.0134 (M^++Na+2H).

2-[(*S*)-1'-Benzyl-2'-oxo-3'-(2''-oxoprop-1''-yl)indolin-3'-yl]malononitrile (4i)

Sticky solid; yield = 86%; $[\alpha]_D^{25}$ = +45 (c 0.1, MeOH); ee = 88% determined by HPLC [Diacel Chiralpak AS-H; hexane/*i*-PrOH 80 : 20; flow rate 1 mL/min; λ = 254 nm; t_R (major) = 10.8 min, t_R (minor) = 16.1 min]; ^1H NMR (300 MHz, $CDCl_3$): δ 2.15 (s, 3H, CH_3), 3.18 (d, J = 17.7 Hz, 1H, CH_2), 3.53 (d, *J* = 17.7 Hz, 1H, CH_2), 4.53 (s, 1H, CH), 5.01 (br s, 2H, CH_2), 6.81 (d, J = 8.1 Hz, 1H, ArH), 7.08-7.17 (m, 2H, ArH), 7.25-7.47 (m, 6H, ArH); ^{13}C NMR (75 MHz, $CDCl_3$): δ 30.1, 30.2, 44.7, 46.5, 48.9, 109.4, 110.4, 110.5, 123.3, 123.6, 124.9, 127.3, 127.8, 128.8, 130.7, 134.6, 143.5, 173.7, 202.5; m/z (ESI-TOF): 366.1236 (M^++Na).

2-[(*S*)-2'-Oxo-3'-(4''-methyl-2''-oxopent-1''-yl)indolin-3'-yl]malononitrile (4j)

Sticky solid; yield = 80%; $[\alpha]_D^{25}$ = +45 (c 0.1, MeOH); ee = 96% determined by HPLC [Diacel Chiralpak IB; hexane/*i*-PrOH 90 : 10; flow rate 1 mL/min; λ = 254 nm; t_R (major) = 27.2 min, t_R (minor) = 16.7 min]; ^1H NMR (300 MHz, $CDCl_3$): δ 0.86 (d, J = 10.5 Hz, 6H, 2 × CH_3), 1.98-2.08 (m, 1H, CH), 2.25-2.28 (m, 2H, CH_2), 3.14 (d, J = 17.7 Hz, 1H, CH_2), 3.45 (d, J = 18.0 Hz, 1H, CH_2), 4.56 (s, 1H, CH), 6.99-7.14 (m, 2H, ArH), 7.33-7.44 (m, 2H, ArH), 8.55 (s, 1H, NH); ^{13}C NMR (75 MHz, $CDCl_3$): δ 14.0, 20.9, 24.6, 49.3, 51.8, 53.7, 60.4, 109.5, 110.4, 111.1, 123.4, 123.6, 125.5, 130.7, 141.5, 171.2, 205.4; m/z (ESI-TOF): 318.1212 (M^++Na).

2-[(*S*)-2'-Oxo-3'-(2''-oxooct-1''-yl)indolin-3'-yl]malononitrile (4k)

Sticky solid; yield = 85%; $[\alpha]_D^{25}$ = +28 (c 0.1, MeOH); ee = 97% determined by HPLC [Diacel Chiralpak AS-H; hexane/*i*-PrOH 90 : 10; flow rate 1 mL/min; λ = 254 nm; t_R (major) = 30.1 min, t_R (minor) = 25.3 min]; ^{1}H NMR (300 MHz, $CDCl_3$): δ 0.84 (t, J = 6.9 Hz, 1H, CH_3), 1.20-1.25 (m, 6H, CH_2), 1.45-1.52 (m, 1H, CH_2), 2.36-2.41 (m, 1H, CH_2), 3.17 (d, J = 18.0 Hz, 1H, CH_2), 3.46 (d, J = 17.4 Hz, 1H, CH_2), 4.58 (s, 1H, CH), 6.99 (d, J = 7.8 Hz, 1H, ArH), 7.08-7.13 (m, 1H, ArH), 7.32-7.43 (m, 2H, ArH), 8.78 (s, 1H, NH); ^{13}C NMR (75 MHz, $CDCl_3$): δ 13.9, 22.4, 23.4, 28.6, 29.9, 30.3, 31.5, 43.1, 45.5, 49.3, 109.7, 110.5, 111.4, 123.6, 125.7, 125.5, 130.8, 141.6, 175.7, 206.1; m/z (ESI-TOF): 346.1518 (M^++Na).

6'-Amino-2'-methyl-2-oxo-2',3'-dihydrospiro[indoline-3,4'-pyran]-5'-carbonitrile (6)

Yellow solid; yield = 90%; m.p. = 283-284 °C; $[\alpha]_D^{25}$ = +28 (c 0.1, MeOH); dr = 82:18; ee = 98% determined by HPLC [Diacel Chiralpak AD-H; hexane/*i*-PrOH 80 : 20; flow rate 1 mL/min; λ = 254 nm; t_R (major) = 13.8 min, t_R (minor) = 12.1 min]; ^{1}H NMR (300 MHz, acetone-d_6): δ 1.32 (d, J = 7.8 Hz, 3H, CH_3), 1.69-1.74 (m, 1H, CH_2), 1.81-1.86 (m, 1H, CH_2), 4.56-4.64 (m, 1H, CH), 5.89 (s, 2H, NH_2), 6.94-7.03 (m, 2H, ArH), 7.20-7.25 (m, 1H, ArH), 7.39 (d, J = 6.0 Hz, 1H, ArH), 9.51 (s, 1H, NH); ^{13}C NMR (75 MHz, acetone-d_6): δ 20.9, 40.0, 48.7, 56.2, 71.8, 110.5, 119.7, 122.8, 124.9, 129.2, 135.8, 141.9, 166.5, 179.8; m/z (ESI-TOF): 278.0915 (M^++Na).

References

[1] For synthesis of isatylidenemalononitriles, see: Demchuk, D. V.; Elinson, M. N.; Nikishin, G. I. *Mendelev Commun.* **2011**, *21*, 224-225.

[2] For synthesis of Primary-tertiary diamines, see: Kumar, A.; Singh, S.; Kumar, V.; Chimni, S. S. *Org. Biomol. Chem.* **2011**, *9*, 2731-2742.

[3] For preparation of spirooxindoles, see: Liu, L.; Wu, D.; Li, X.; Wang, S.; Li, H.; Li, J.; Wang, W. *Chem. Commun.* **2012**, *48*, 1692-1694;

C:¥Users¥HP¥Desktop¥other¥AKS_NMR¥19-5-2012¥Dr_Chimni¥ C-AKS3211NON_E3_FT.als

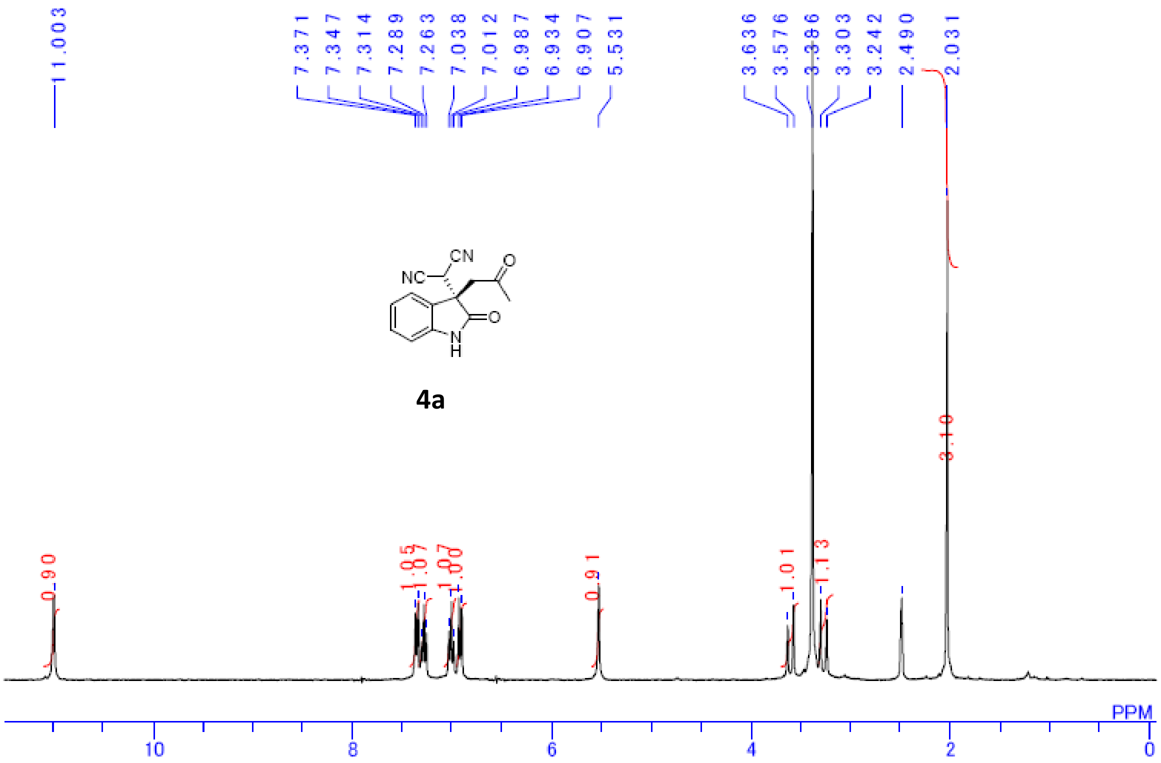

C:¥Users¥HP¥Desktop¥other¥AKS_NMR¥19-5-2012¥Dr_Chimni¥AKS322.als

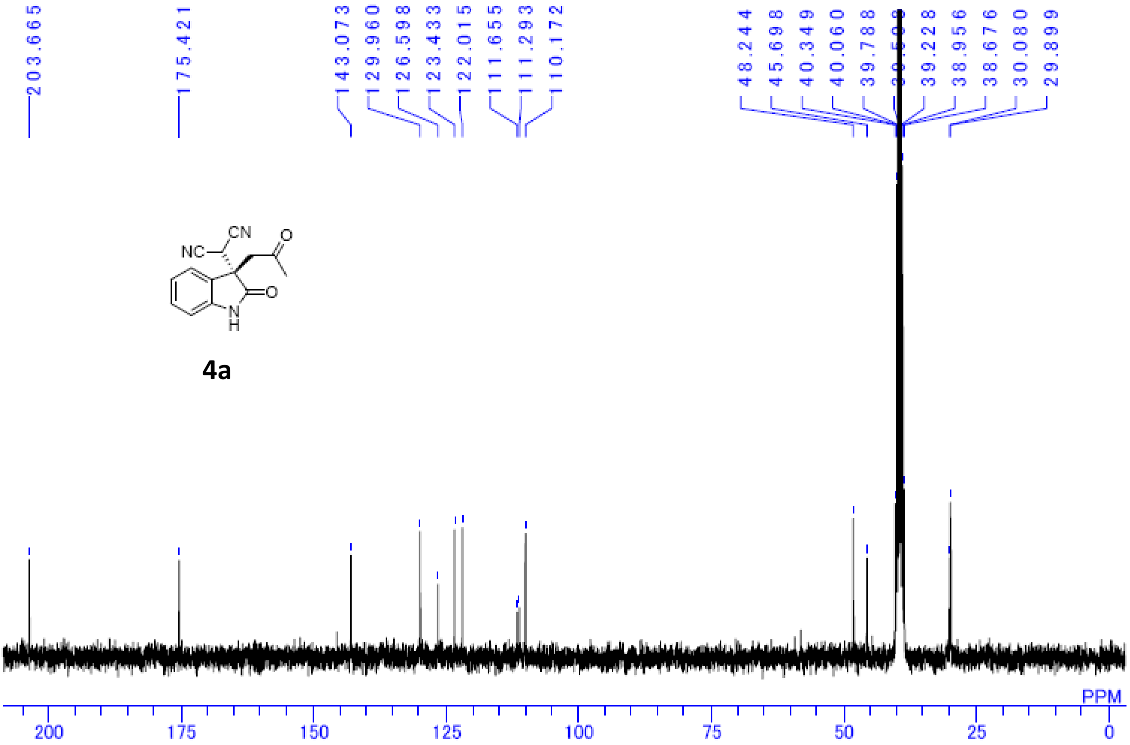

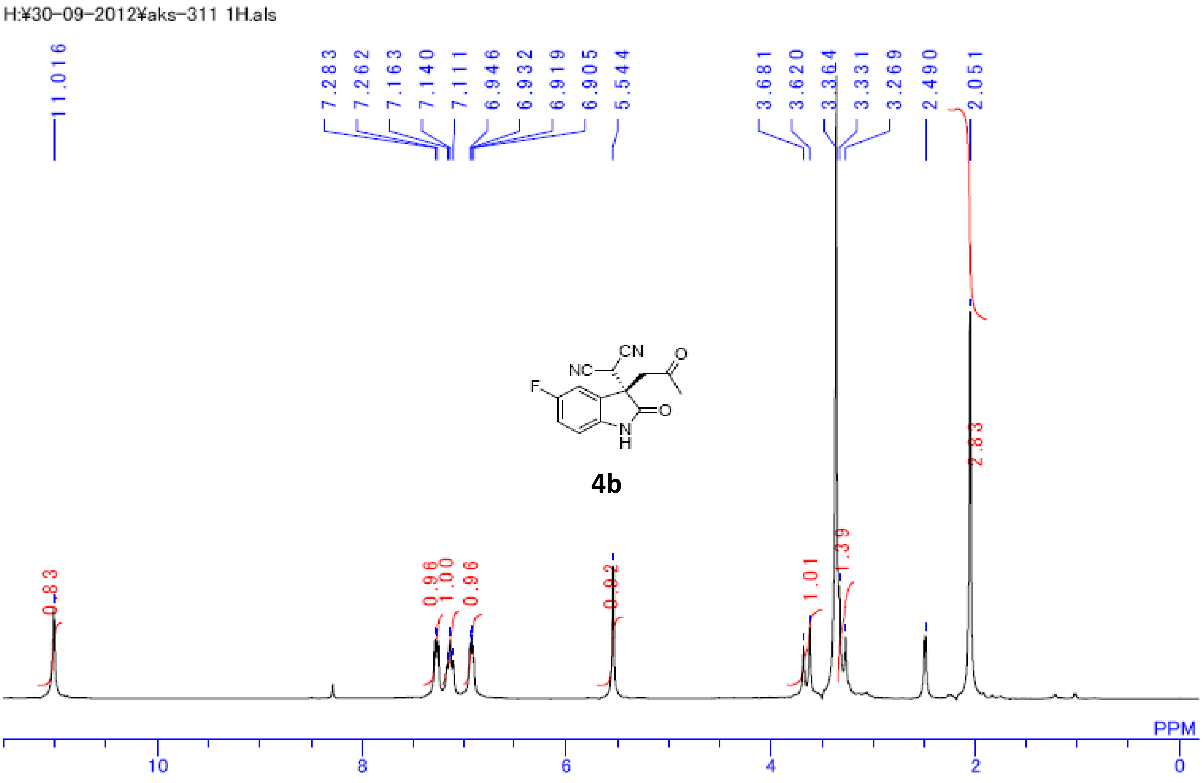
H:¥30-09-2012¥aks-311 1H.als
11.016
7.283
7.262
7.163
7.140
7.111
6.946
6.932
6.919
6.905
5.544
3.681
3.620
3.364
3.331
3.269
2.490
2.051
NC
CN
O
F
O
N
H
0.83
0.96
1.00
0.96
0.92
1.01
1.39
2.83
PPM
10
8
6
4
2
0

4b

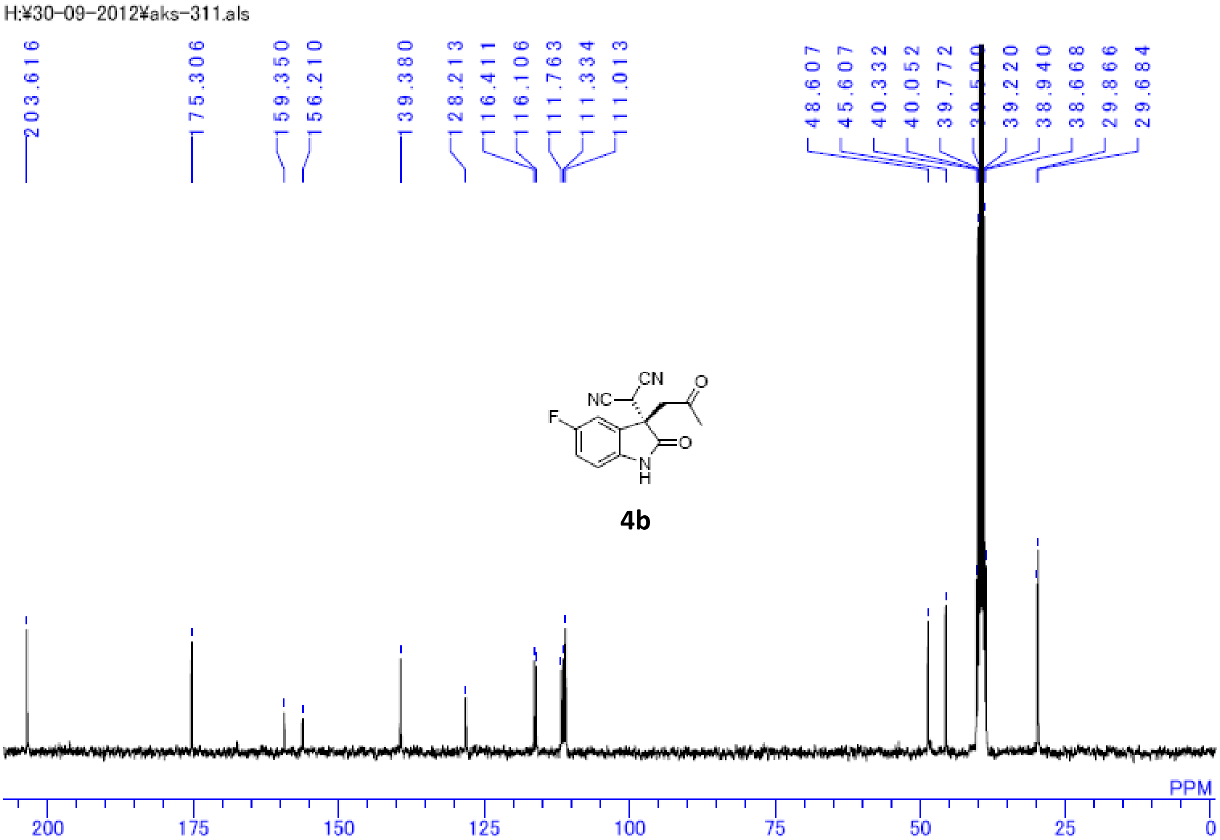
H:¥30-09-2012¥aks-311.als
203.616
175.306
159.350
156.210
139.380
128.213
116.411
116.106
111.763
111.334
111.013
48.607
45.607
40.332
40.052
39.772
39.220
38.940
38.668
29.866
29.684
NC
CN
O
F
O
N
H
PPM
200
175
150
125
100
75
50
25
0

4b

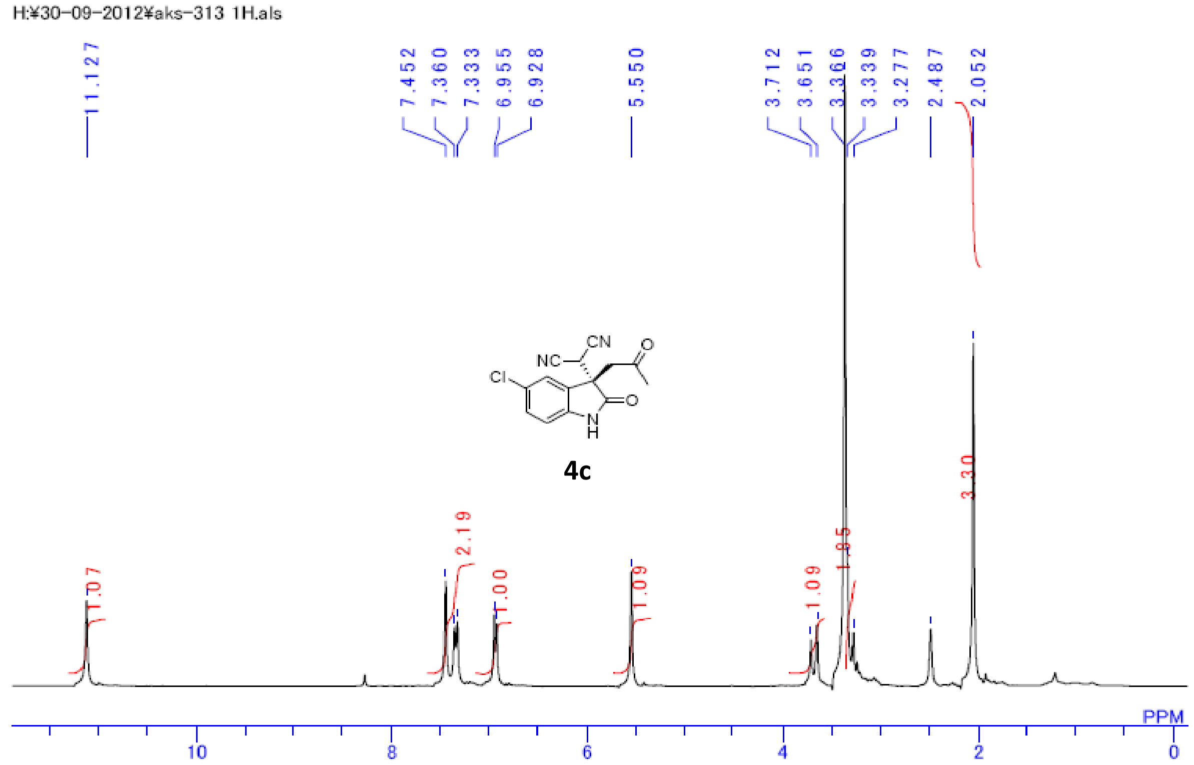
H:¥30-09-2012¥aks-313 1H.als
11.127
7.452
7.360
7.333
6.955
6.928
5.550
3.712
3.651
3.366
3.339
3.277
2.487
2.052
NC
CN
O
Cl
O
N
H
4c
1.07
2.19
1.00
1.09
1.09
1.95
3.30
PPM
10
8
6
4
2
0

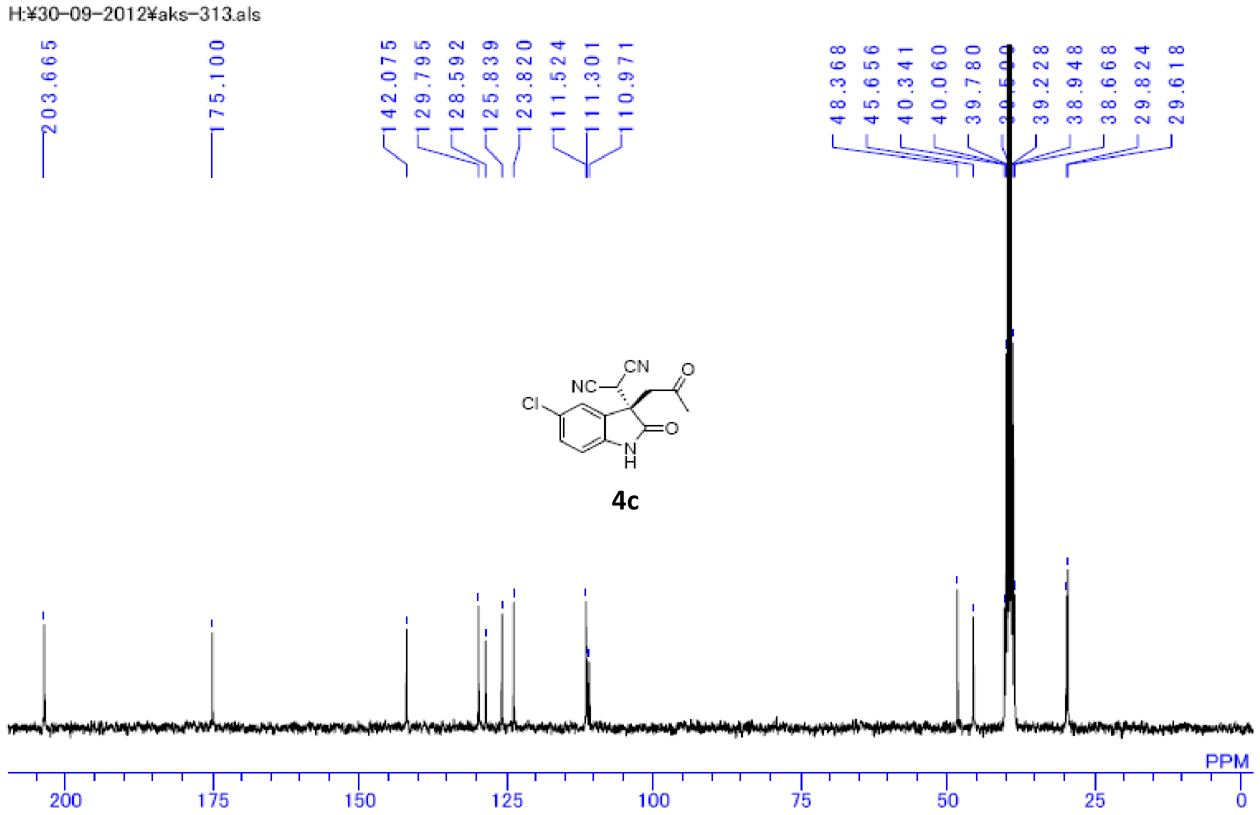
H:¥30-09-2012¥aks-313.als
203.665
175.100
142.075
129.795
128.592
125.839
123.820
111.524
111.301
110.971
48.368
45.656
40.341
40.060
39.780
39.228
38.948
38.668
29.824
29.618
NC
CN
O
Cl
O
N
H
4c
PPM
200
175
150
125
100
75
50
25
0

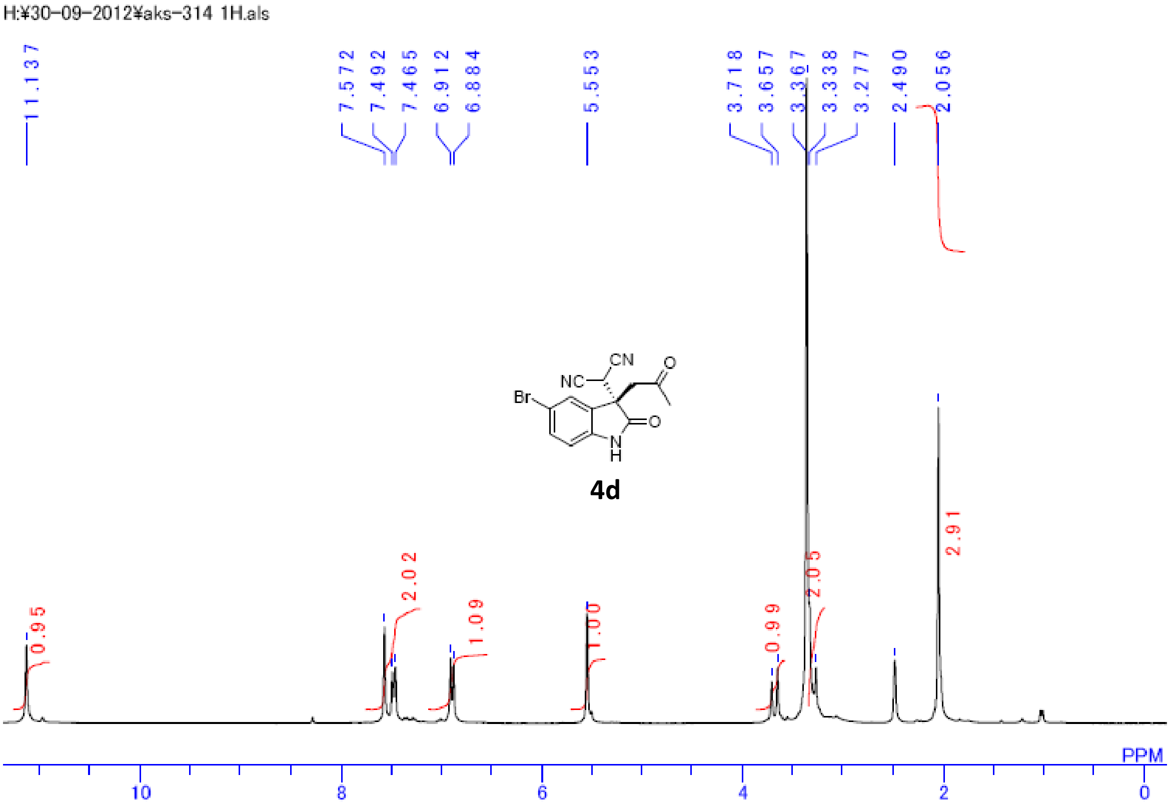
H:¥30-09-2012¥aks-314 1H.als
11.137
7.572
7.492
7.465
6.912
6.884
5.553
3.718
3.657
3.367
3.338
3.277
2.490
2.056
0.95
2.02
1.09
1.00
0.99
2.05
2.91
Br
NC
CN
O
N
H
4d
PPM
10
8
6
4
2
0

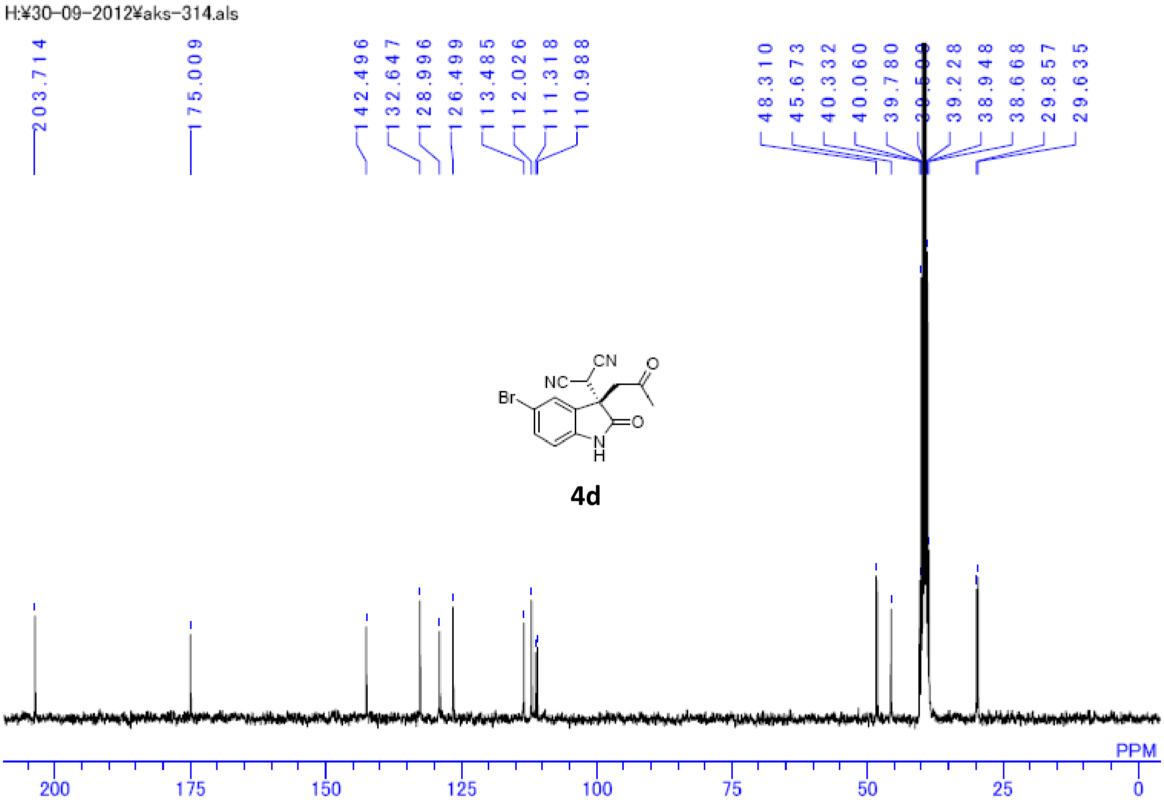
H:¥30-09-2012¥aks-314.als
203.714
175.009
142.496
132.647
128.996
126.499
113.485
112.026
111.318
110.988
48.310
45.673
40.332
40.060
39.780
39.228
38.948
38.668
29.857
29.635
Br
NC
CN
O
N
H
4d
PPM
200
175
150
125
100
75
50
25
0

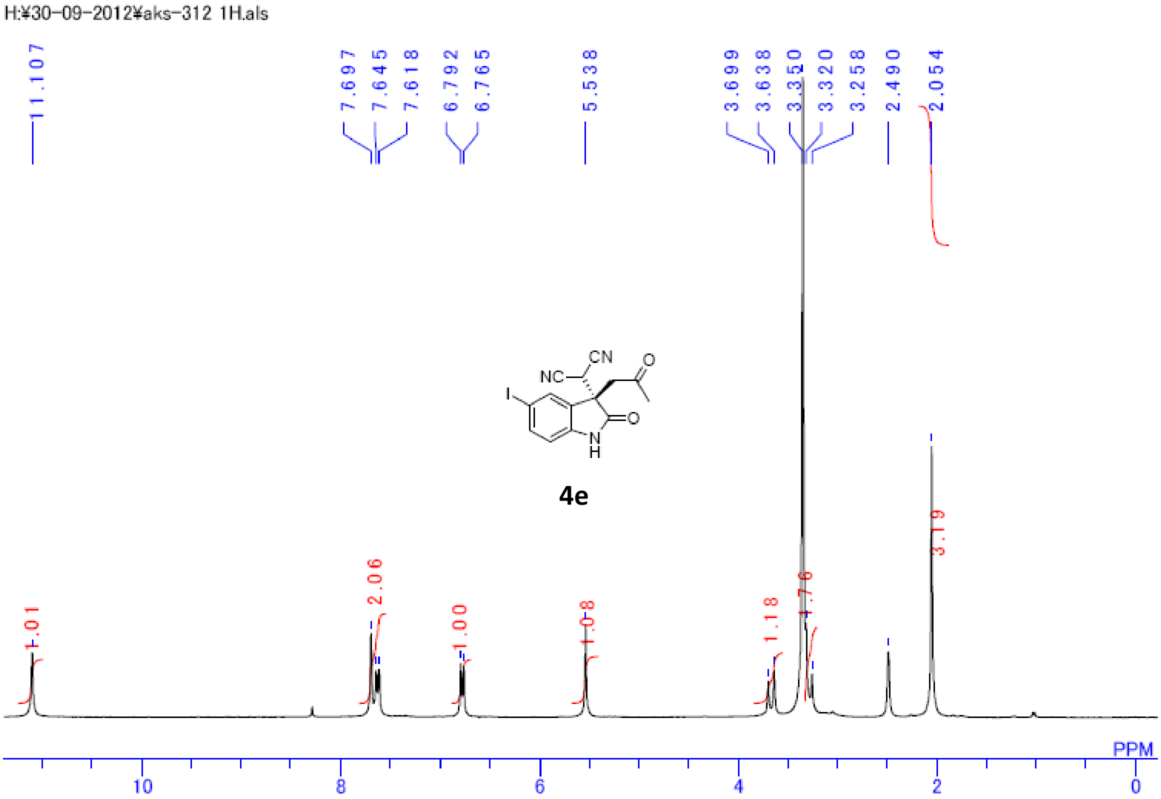
H:¥30-09-2012¥aks-312 1H.als
11.107
7.697
7.645
7.618
6.792
6.765
5.538
3.699
3.638
3.350
3.320
3.258
2.490
2.054
1.01
2.06
1.00
1.18
3.19
NC
CN
O
I
O
N
H
4e
PPM
10
8
6
4
2
0

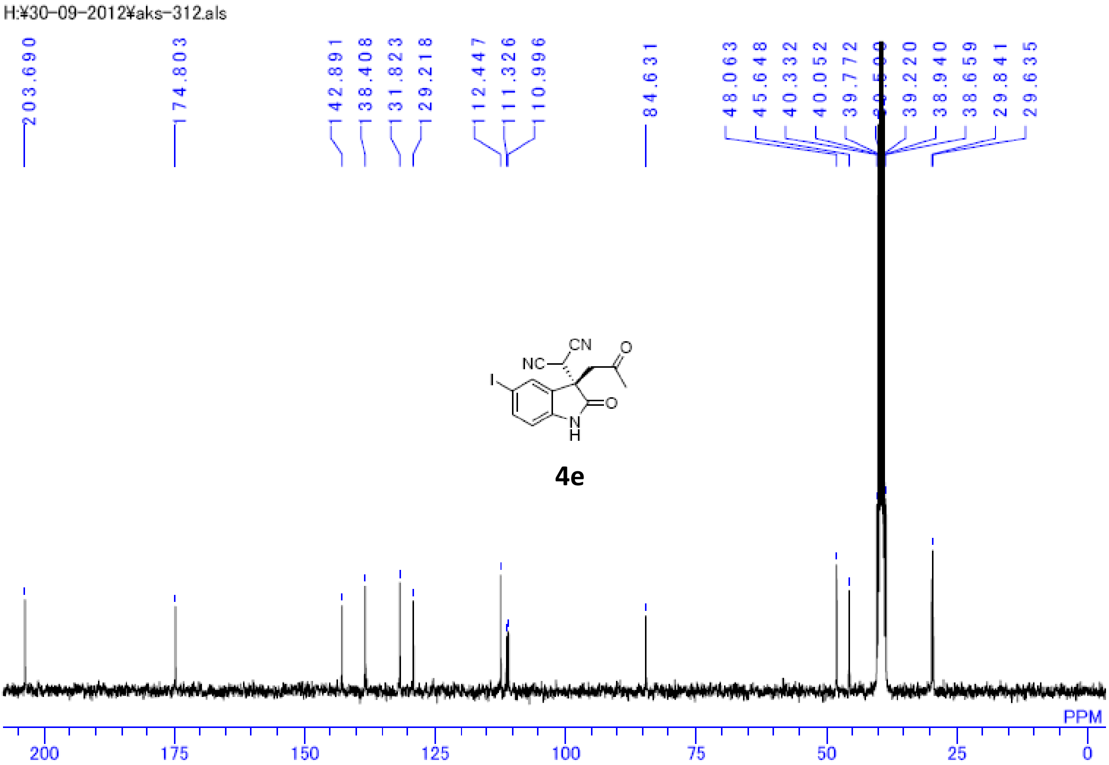
H:¥30-09-2012¥aks-312.als
203.690
174.803
142.891
138.408
131.823
129.218
112.447
111.326
110.996
84.631
48.063
45.648
40.332
40.052
39.772
39.220
38.940
38.659
29.841
29.635
NC
CN
O
I
O
N
H
4e
PPM
200
175
150
125
100
75
50
25
0

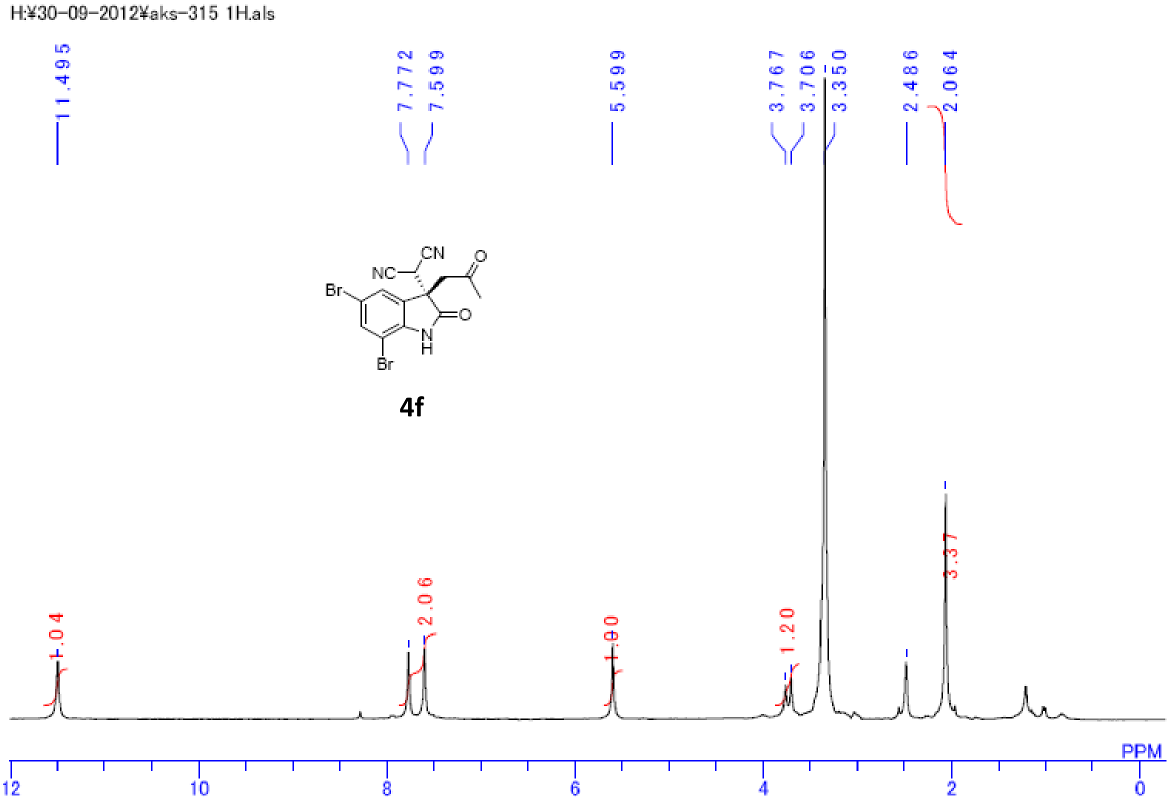
H:¥30-09-2012¥aks-315 1H.als
11.495
7.772
7.599
5.599
3.767
3.706
3.350
2.486
2.064
NC
CN
O
Br
N
H
Br
4f
1.04
2.06
1.00
1.20
3.37
PPM
12
10
8
6
4
2
0

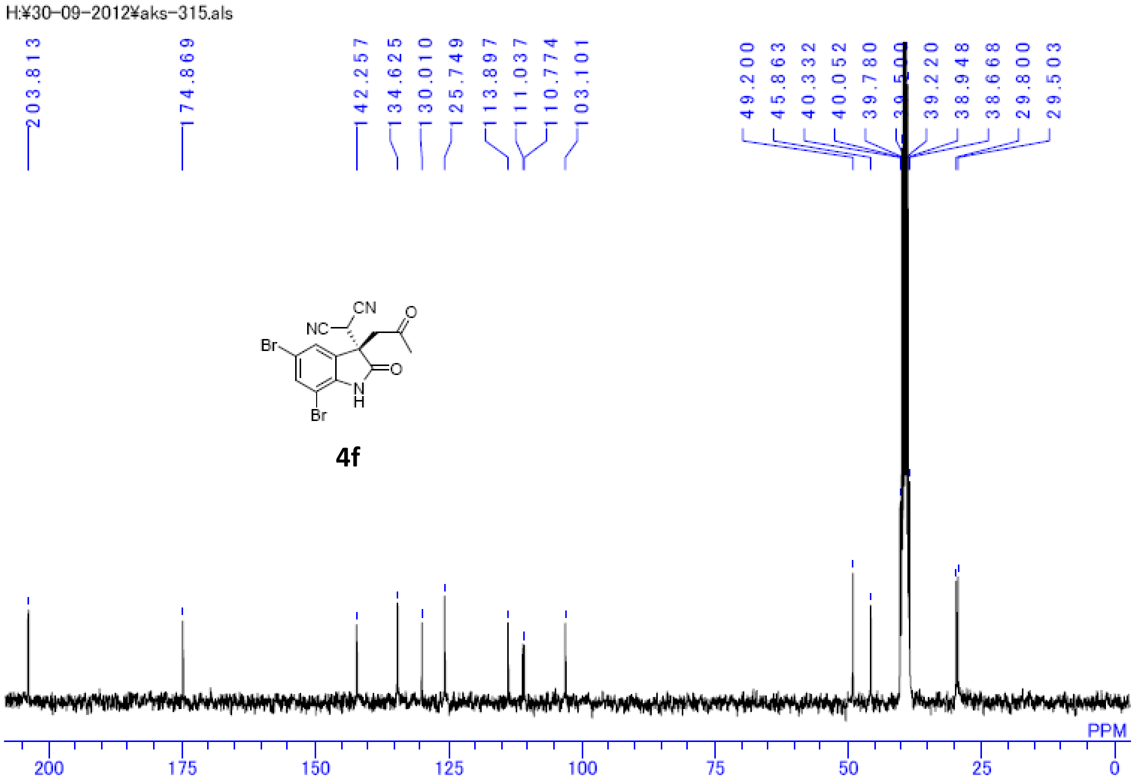
H:¥30-09-2012¥aks-315.als
203.813
174.869
142.257
134.625
130.010
125.749
113.897
111.037
110.774
103.101
49.200
45.863
40.332
40.052
39.780
39.220
38.948
38.668
29.800
29.503
NC
CN
O
Br
N
H
Br
4f
PPM
200
175
150
125
100
75
50
25
0

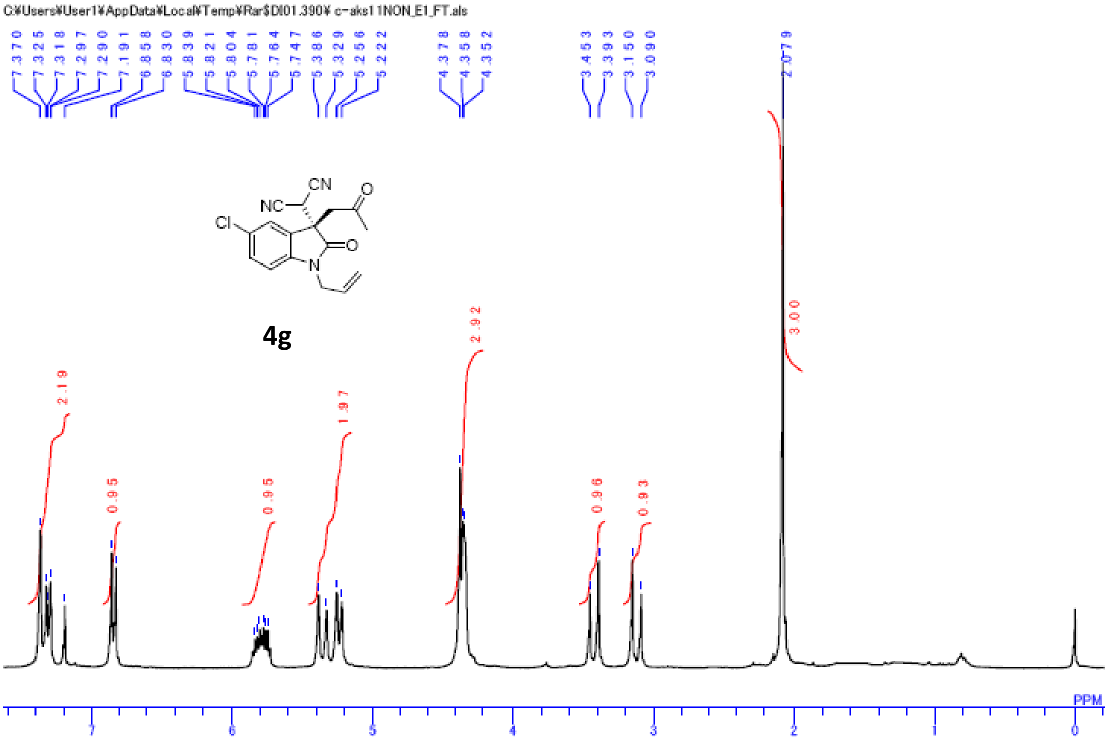
C:¥Users¥User1¥AppData¥Local¥Temp¥Rar$DI01.390¥ c-aks11NON_E1_FT.als
7.370
7.325
7.318
7.297
7.290
7.191
6.858
6.830
5.839
5.821
5.804
5.781
5.764
5.747
5.386
5.329
5.256
5.222
4.378
4.358
4.352
3.453
3.393
3.150
3.090
2.079
2.19
0.95
0.95
1.97
2.92
0.96
0.93
3.00
CN
NC
O
Cl
N
O
4g
PPM
7
6
5
4
3
2
0

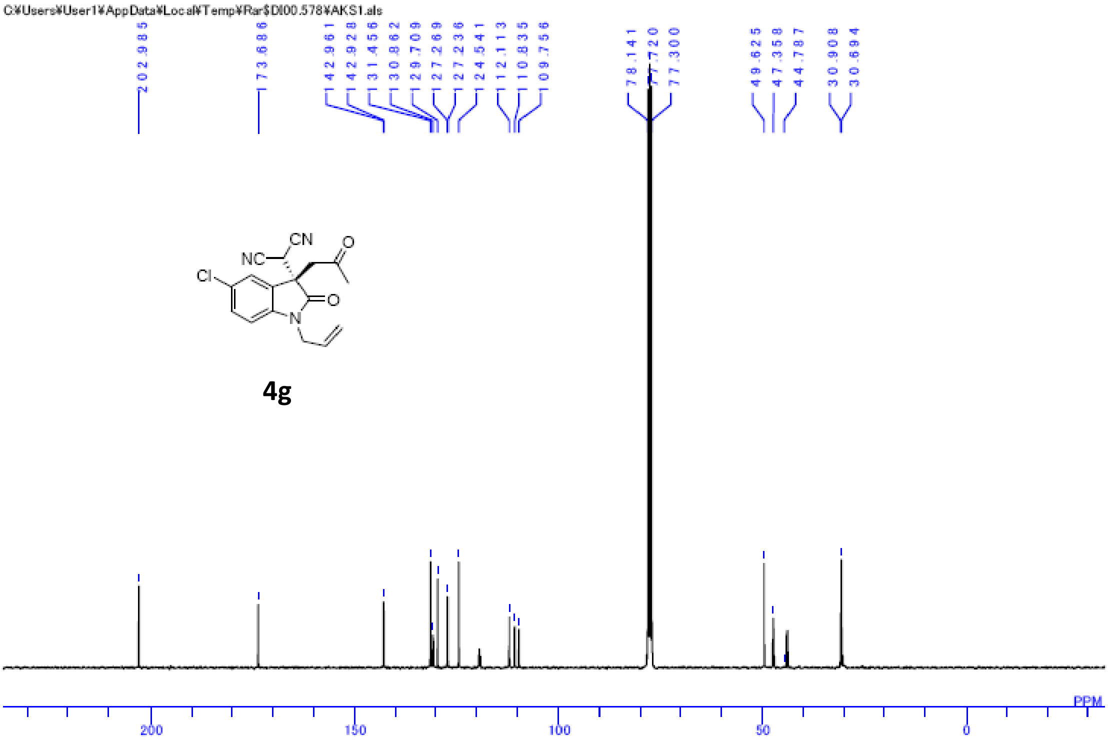
C:¥Users¥User1¥AppData¥Local¥Temp¥Rar$DI00.578¥AKS1.als
202.985
173.686
142.961
142.928
131.456
130.862
129.709
127.269
127.236
124.541
112.113
110.835
109.756
78.141
77.720
77.300
49.625
47.358
44.787
30.908
30.694
CN
NC
O
Cl
N
O
4g
PPM
200
150
100
50
0

C:¥Users¥HP¥Desktop¥other¥AKS_NMR¥aks¥Dr_Chimni¥Dr_Chimni¥ C-AKS3081NON_E1_FT.als

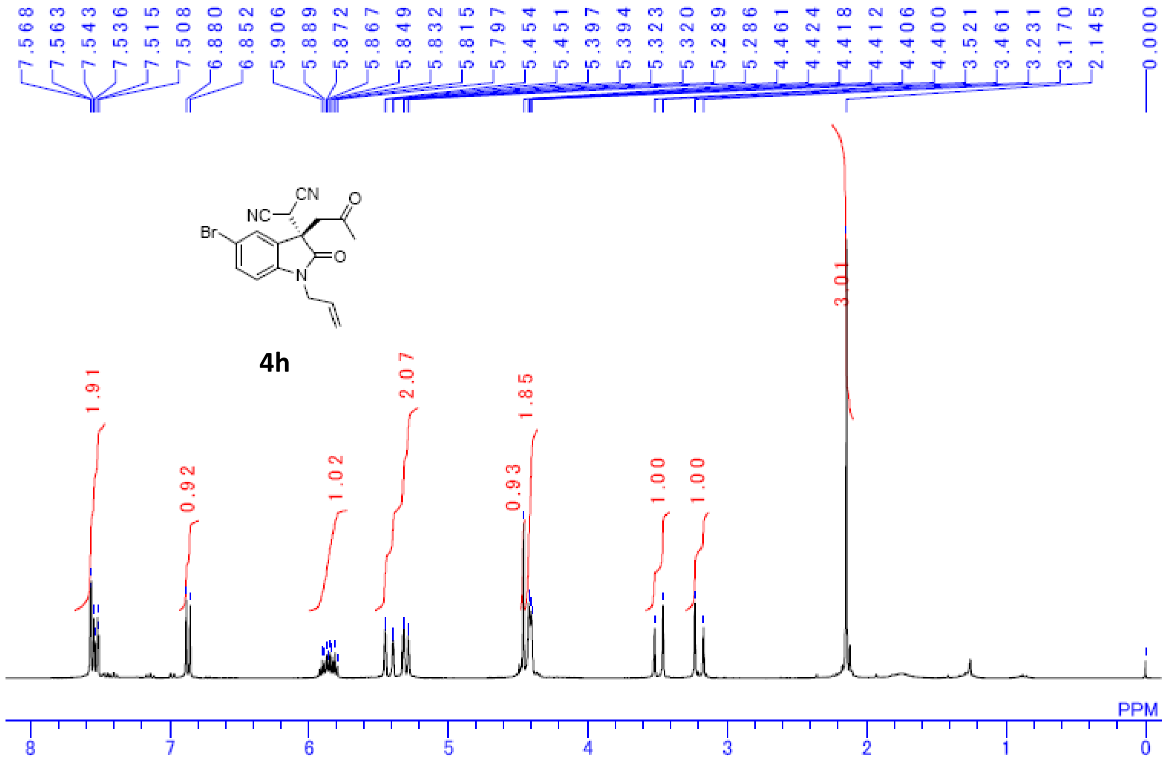

C:¥Users¥HP¥Desktop¥other¥AKS_NMR¥aks¥Dr_Chimni¥Dr_Chimni¥ C-AKS3051BCM_E4_FT.als

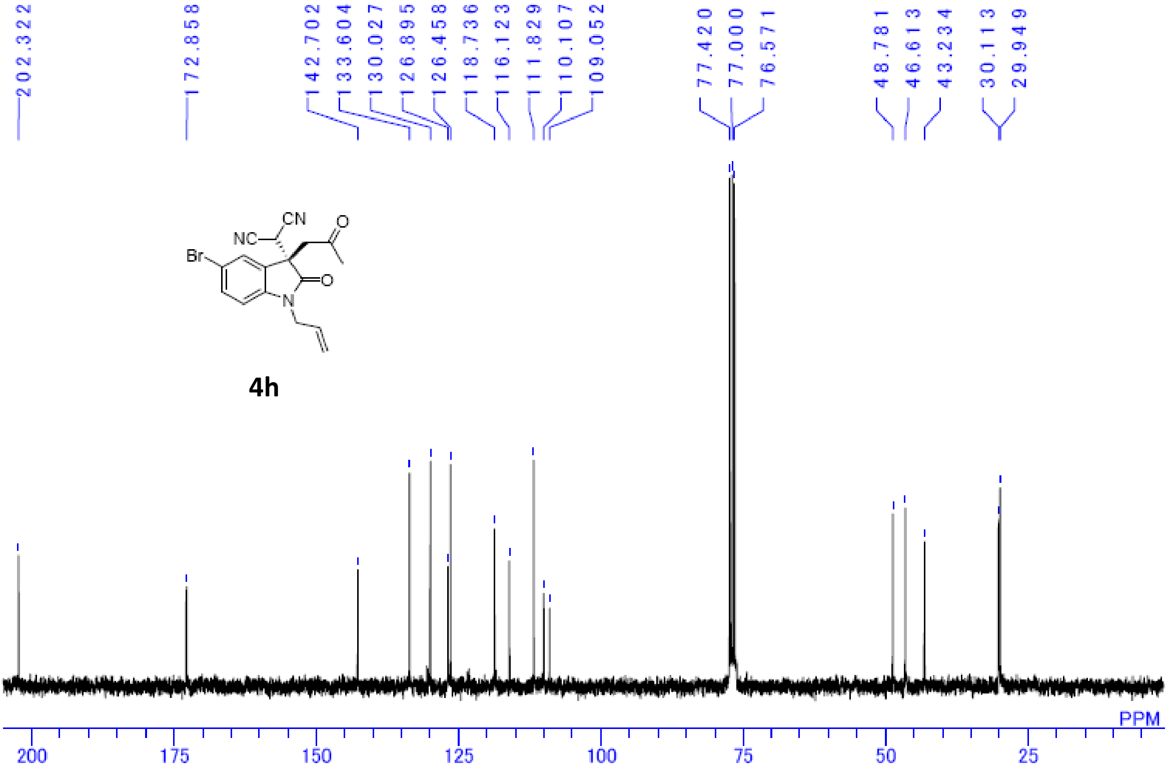

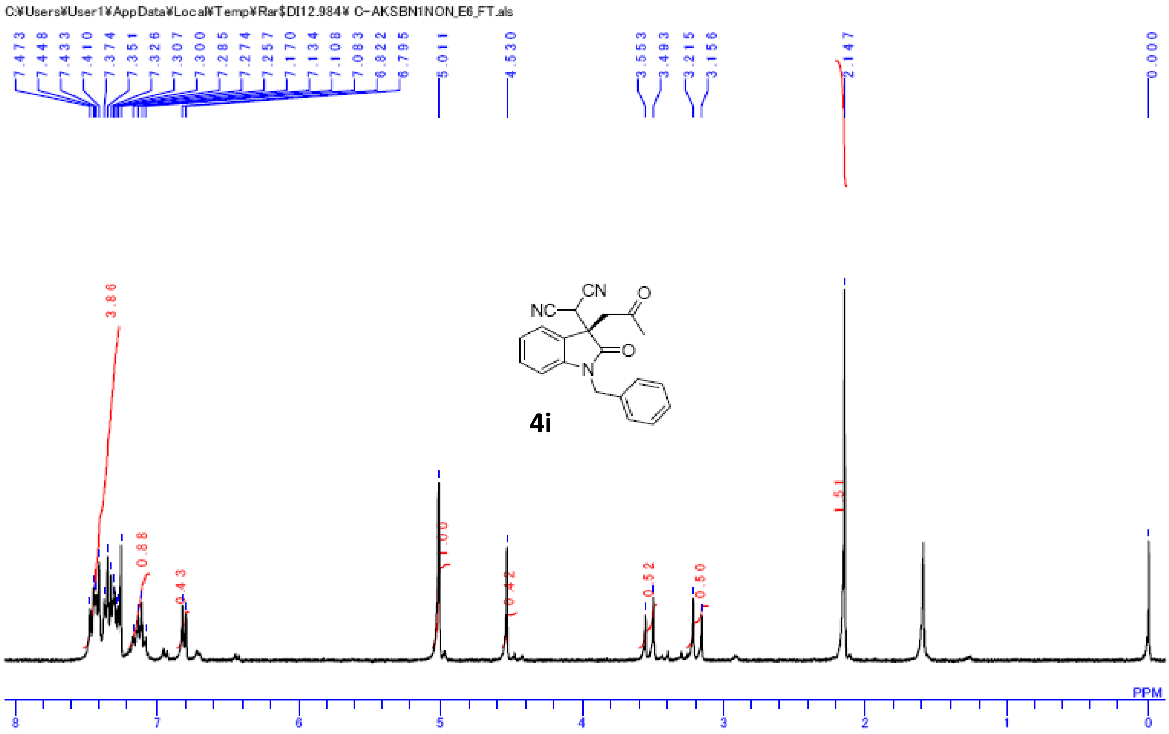
C:¥Users¥User1¥AppData¥Local¥Temp¥Rar$DI12.984¥ C-AKSBN1NON_E6_FT.als
7.473 7.448 7.433 7.410 7.374 7.351 7.326 7.307 7.300 7.285 7.274 7.257 7.170 7.134 7.108 7.083 6.822 6.795 5.011 4.530 3.553 3.493 3.215 3.156 2.147 0.000
3.86 0.88 0.43 1.00 0.42 0.52 0.50 1.51
NC
CN
O
N
4i
PPM

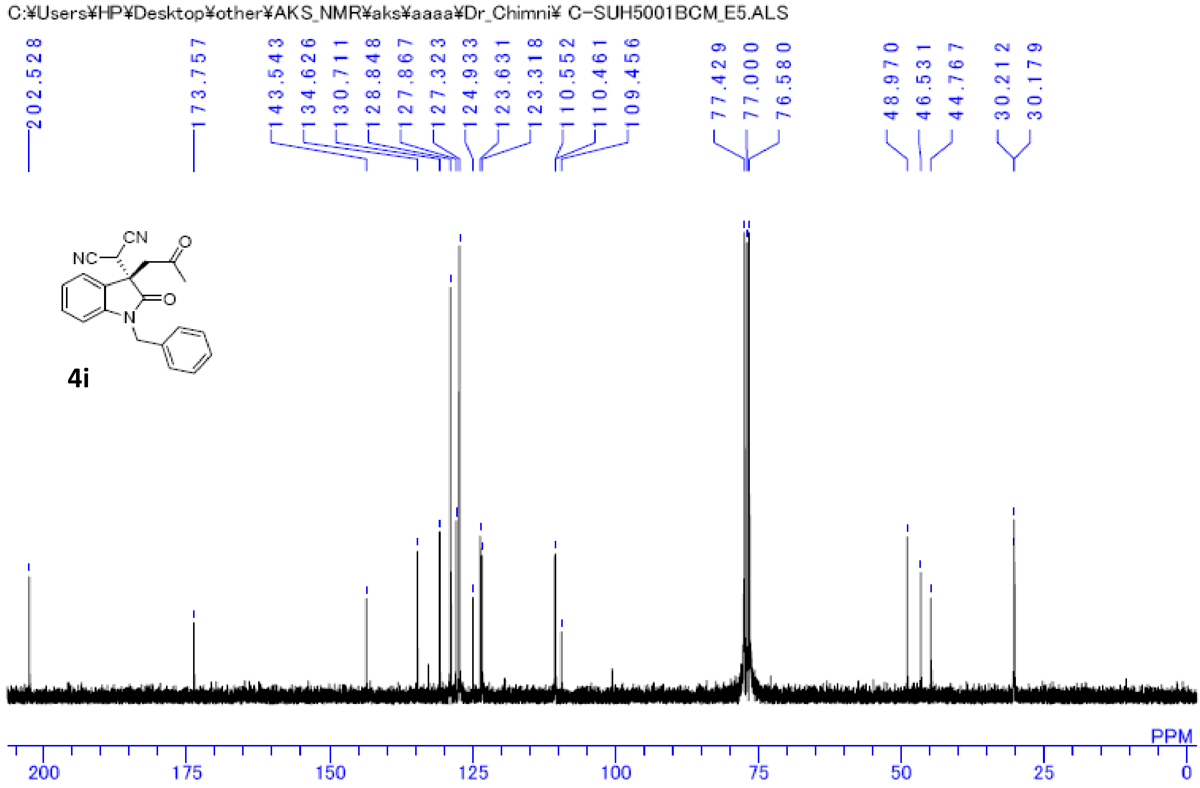
C:¥Users¥HP¥Desktop¥other¥AKS_NMR¥aks¥aaaa¥Dr_Chimni¥ C-SUH5001BCM_E5.ALS
202.528 173.757 143.543 134.626 130.711 128.848 127.867 127.323 124.933 123.631 123.318 110.552 110.461 109.456 77.429 77.000 76.580 48.970 46.531 44.767 30.212 30.179
NC
CN
O
N
4i
PPM

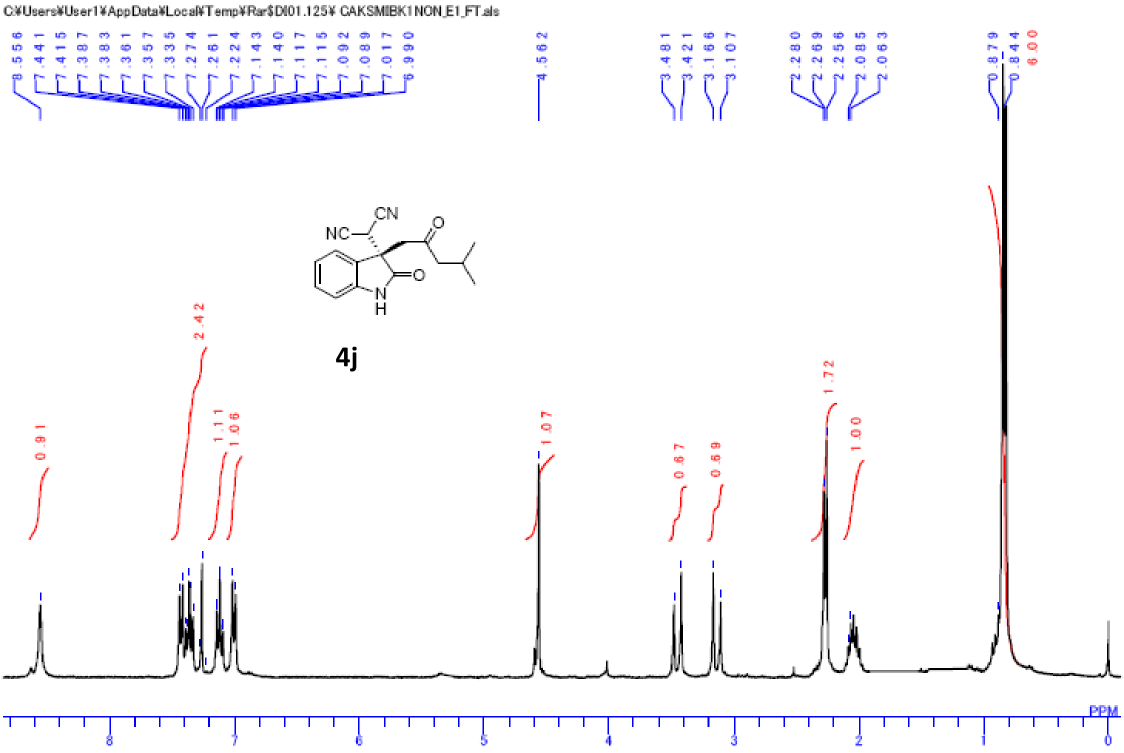
C:¥Users¥User1¥AppData¥Local¥Temp¥Rar$DI01.125¥ CAKSMIBK1NON_E1_FT.als
4j
PPM

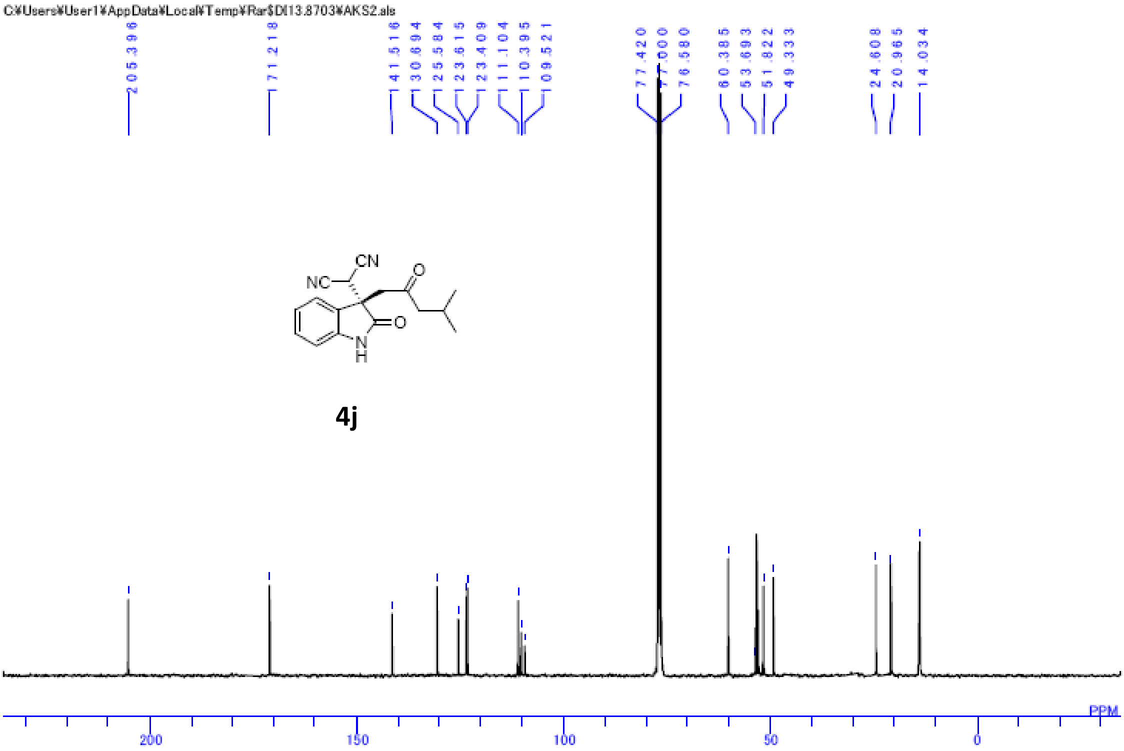
C:¥Users¥User1¥AppData¥Local¥Temp¥Rar$DI13.8703¥AKS2.als
4j
PPM

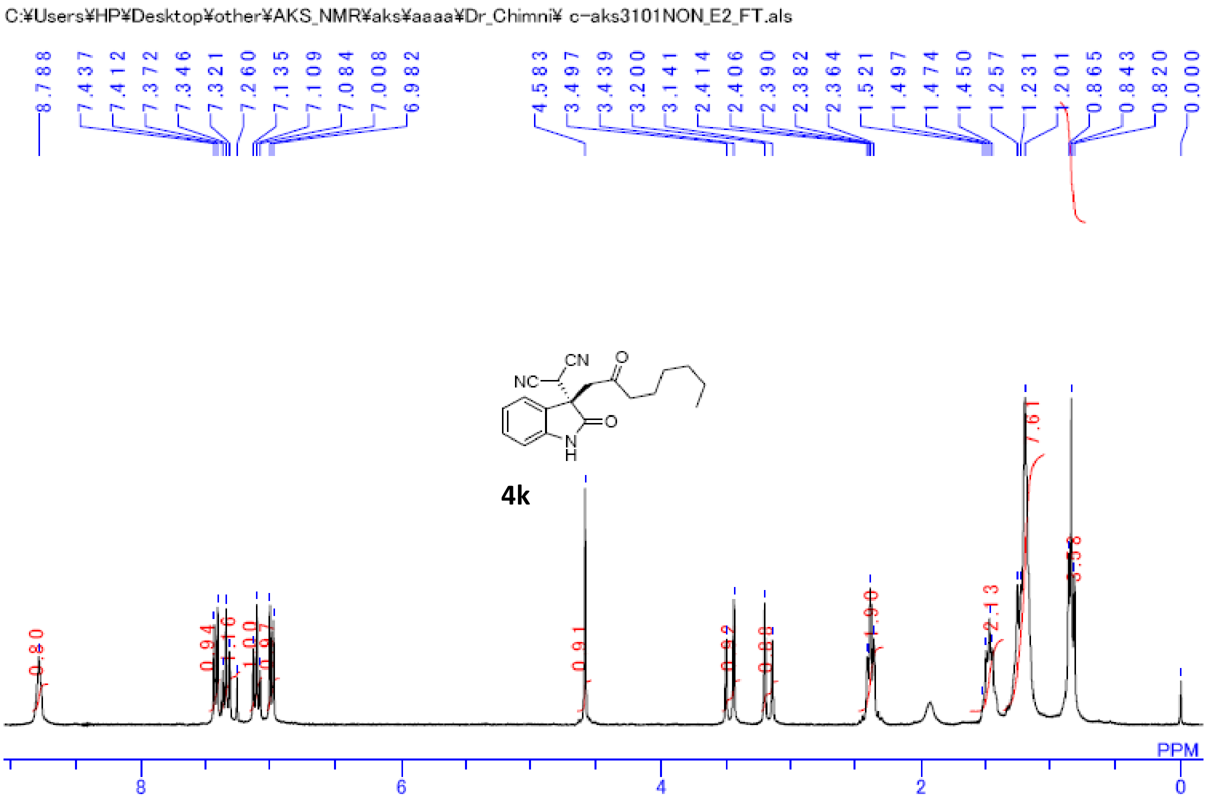
C:¥Users¥HP¥Desktop¥other¥AKS_NMR¥aks¥aaaa¥Dr_Chimni¥ c-aks3101NON_E2_FT.als
8.788
7.437
7.412
7.372
7.346
7.321
7.260
7.135
7.109
7.084
7.008
6.982
4.583
3.497
3.439
3.200
3.141
2.414
2.406
2.390
2.382
2.364
1.521
1.497
1.474
1.450
1.257
1.231
1.201
0.865
0.843
0.820
0.000
NC
CN
O
O
N
H
4k
PPM
8
6
4
2
0

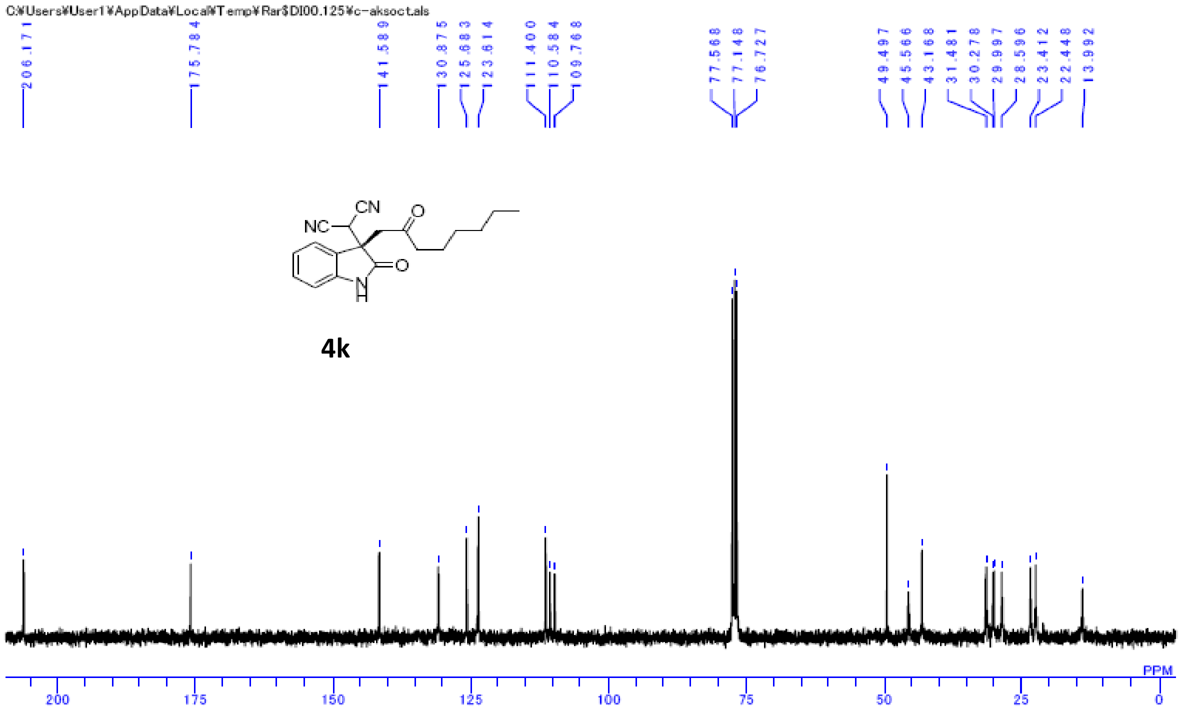
C:¥Users¥User1¥AppData¥Local¥Temp¥Rar$DI00.125¥c-aksoct.als
206.171
175.784
141.589
130.875
125.683
123.614
111.400
110.584
109.768
77.568
77.148
76.727
49.497
45.566
43.168
31.481
30.278
29.997
28.596
23.412
22.448
13.992
NC
CN
O
O
N
H
4k
PPM
200
175
150
125
100
75
50
25
0

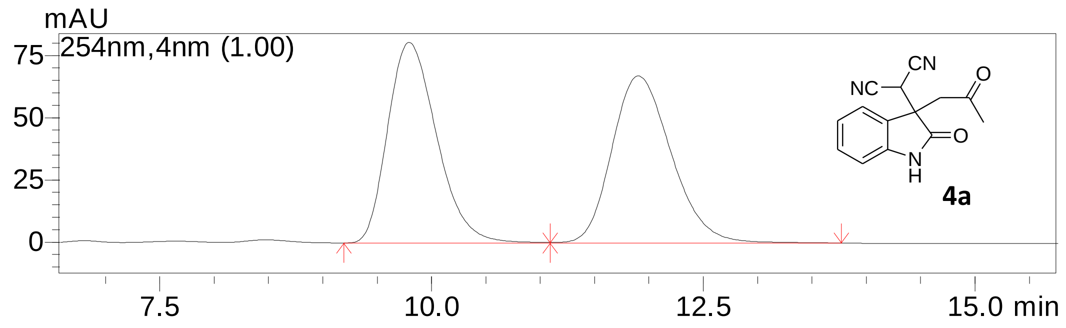

Entry	Ret. Time	Area%
1	9.788	49.7678
2	11.898	50.2322

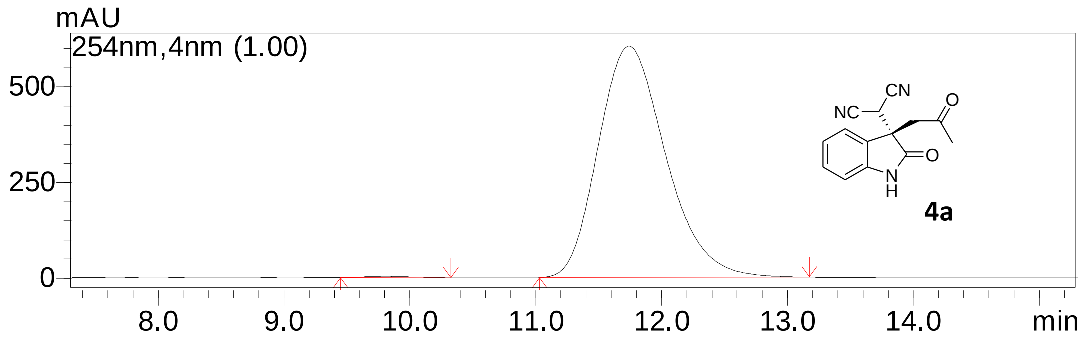

Entry	Ret. Time	Area%
1	9.806	0.3825
2	11.735	99.6175

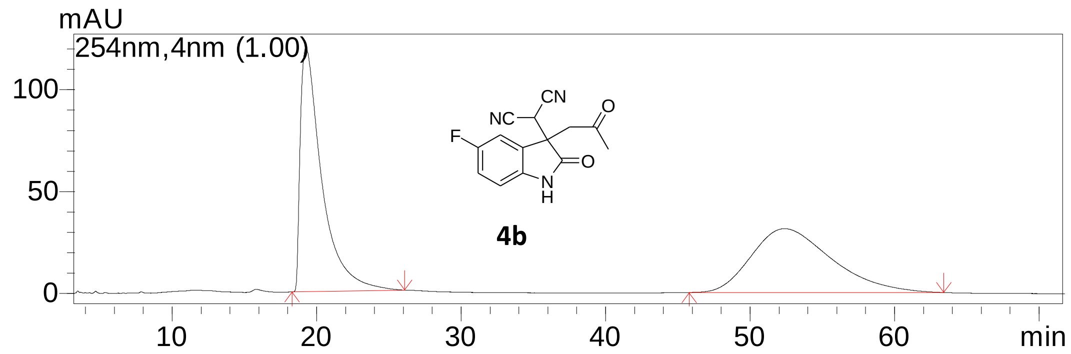

Peak	Ret. Time	Area%
1	19.252	50.6597
2	52.381	49.3403

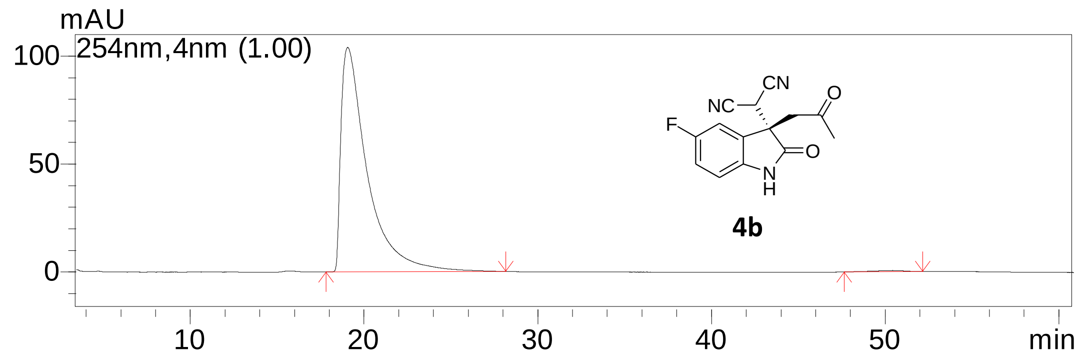

Peak	Ret. Time	Area%
1	19.052	99.3137
2	50.426	0.6863

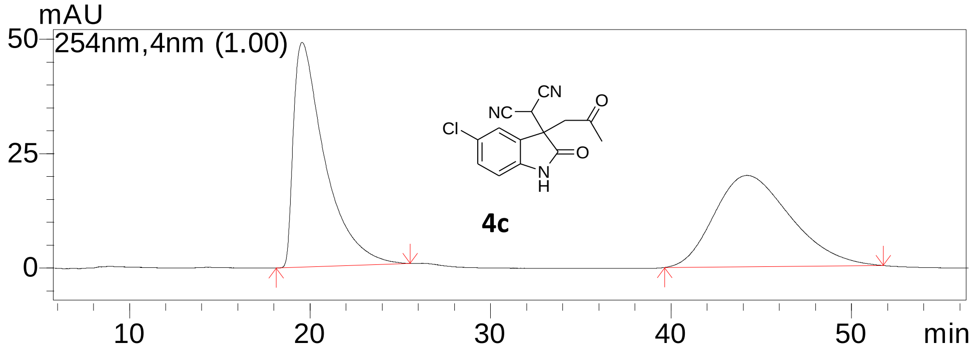

Peak	Ret. Time	Area%
1	19.551	50.2963
2	44.227	49.7037

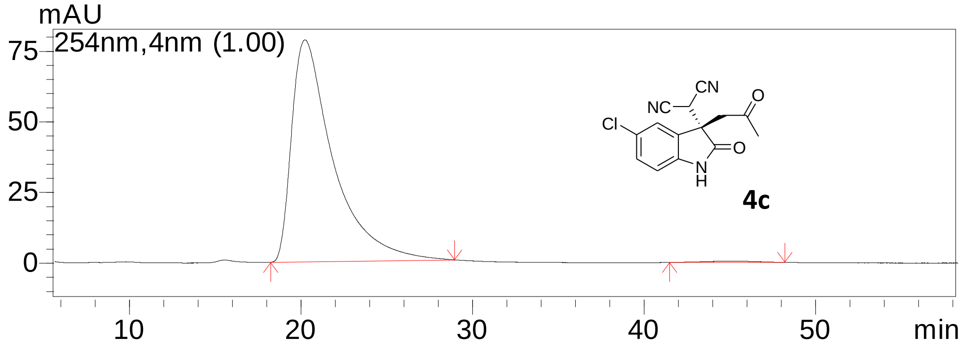

Peak	Ret. Time	Area%
1	20.227	99.2792
2	45.109	0.7208

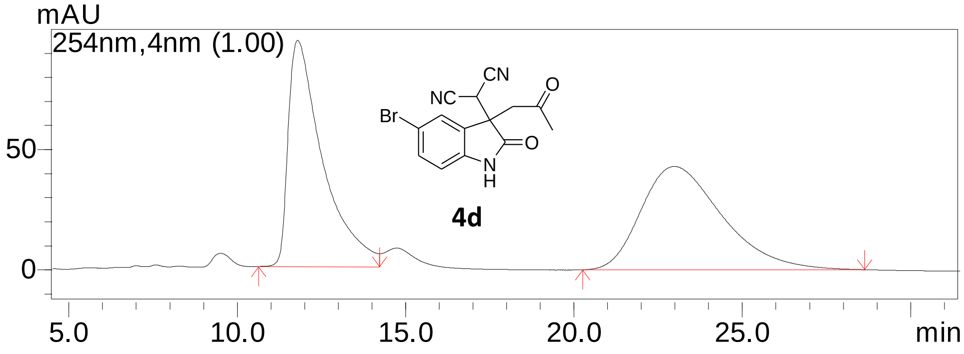

Peak	Ret. Time	Area%
1	11.791	48.3476
2	22.964	51.6524

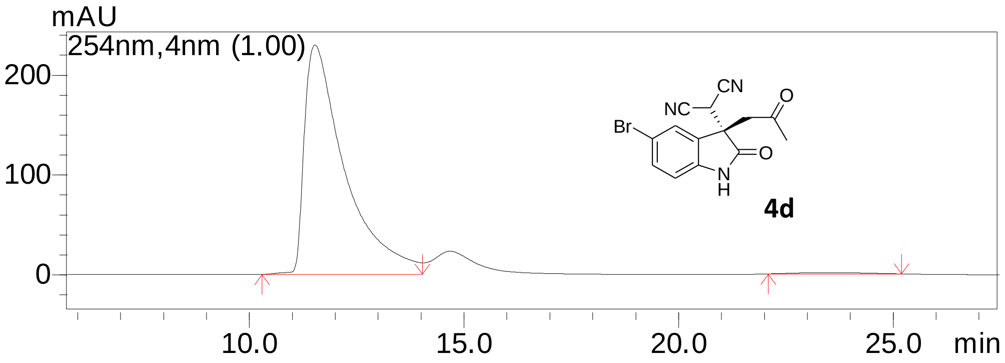

Peak	Ret. Time	Area%
1	11.520	99.0534
2	23.524	0.9466

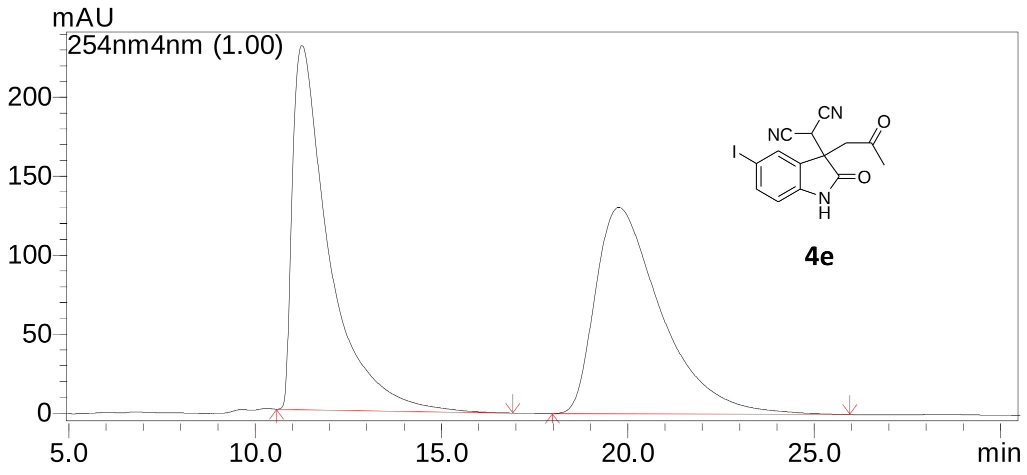

Peak	Ret. Time	Area%
1	11.251	49.4420
2	19.750	50.5580

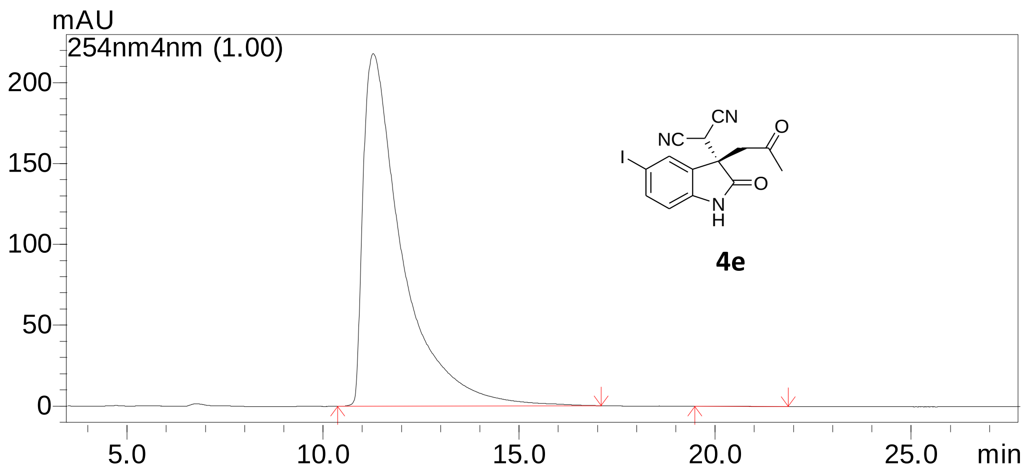

Peak	Ret. Time	Area%
1	11.276	99.9526
2	20.294	0.0474

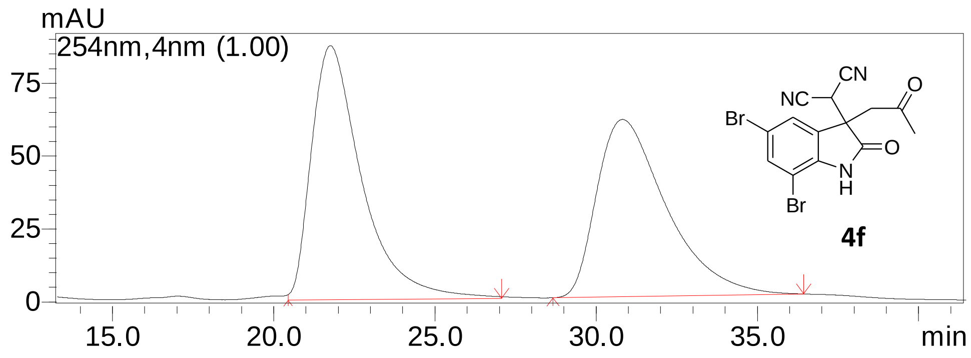

Peak	Ret. Time	Area%
1	21.753	50.9149
2	30.820	49.0851

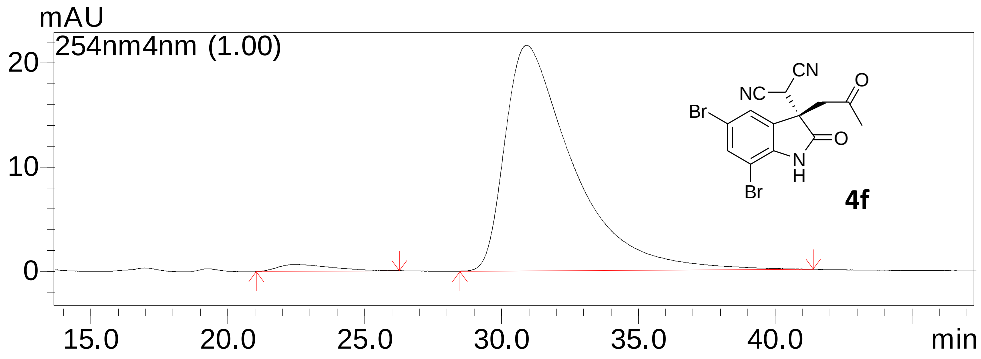

Peak	Ret. Time	Area%
1	22.451	2.3424
2	30.894	97.6576

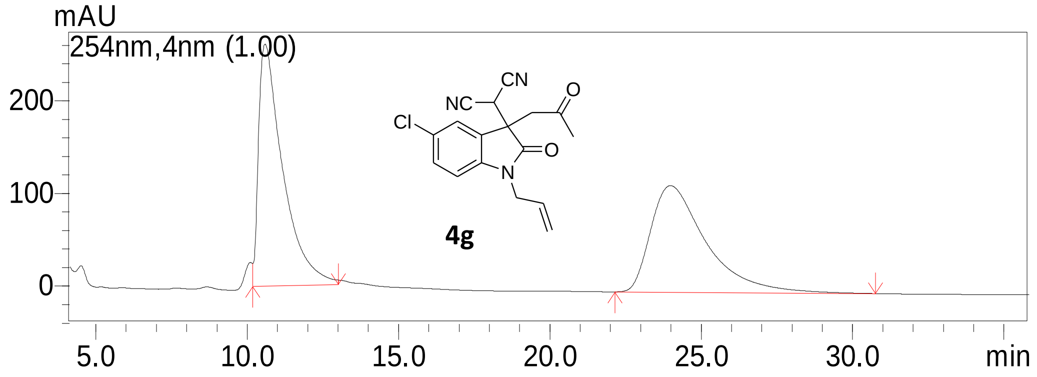

Peak	Ret. Time	Area%
d1	10.585	49.9527
2	23.983	50.0473

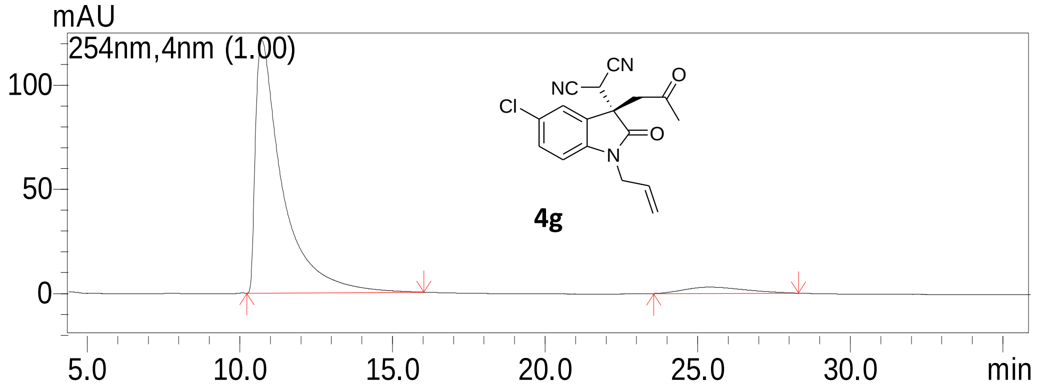

Peak	Ret. Time	Area%
1	10.719	94.6486
2	25.426	5.3514

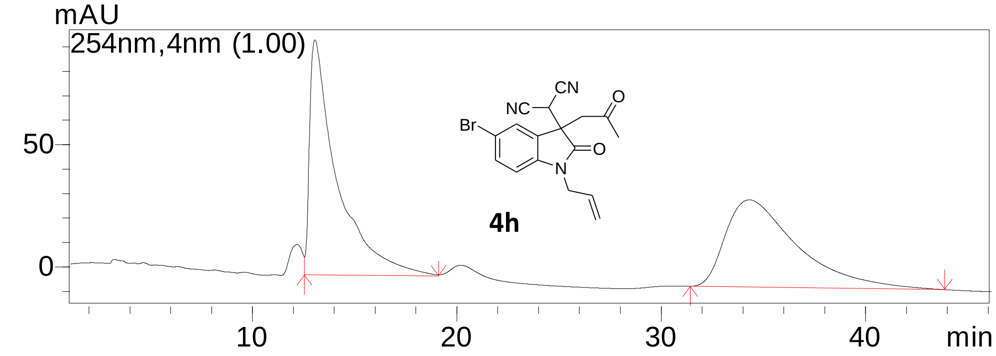

Peak	Ret. Time	Area%
1	13.047	50.7389
2	34.298	49.2611

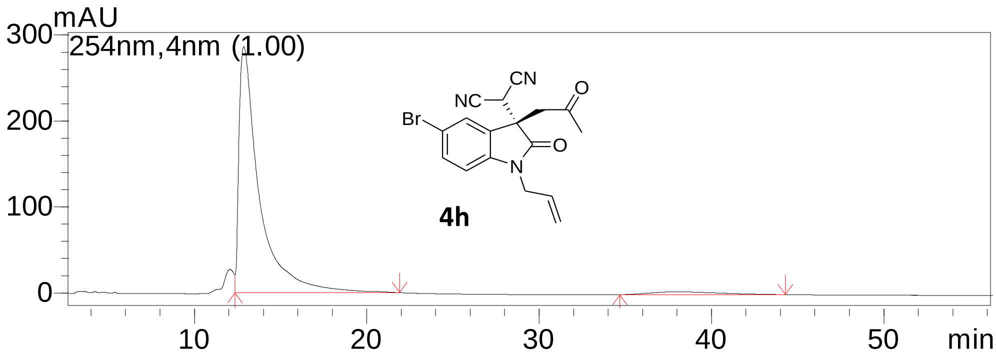

Peak	Ret. Time	Area%
1	12.860	96.2548
2	37.916	3.7452

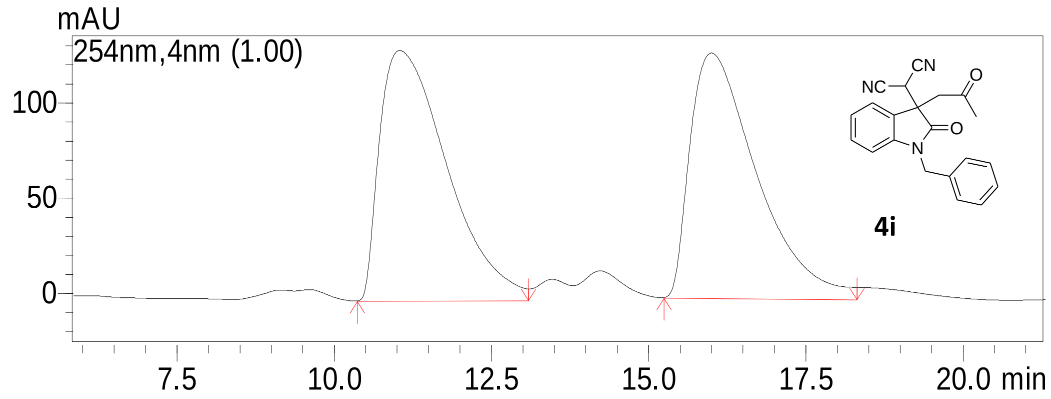

Peak	Ret. Time	Area%
1	11.036	50.6657
2	15.994	49.3343

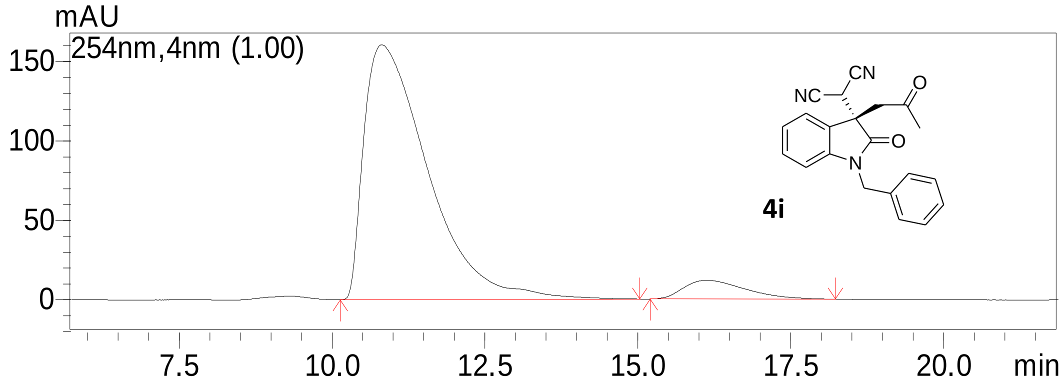

Peak	Ret. Time	Area%
1	10.808	93.3538
2	16.129	6.6462

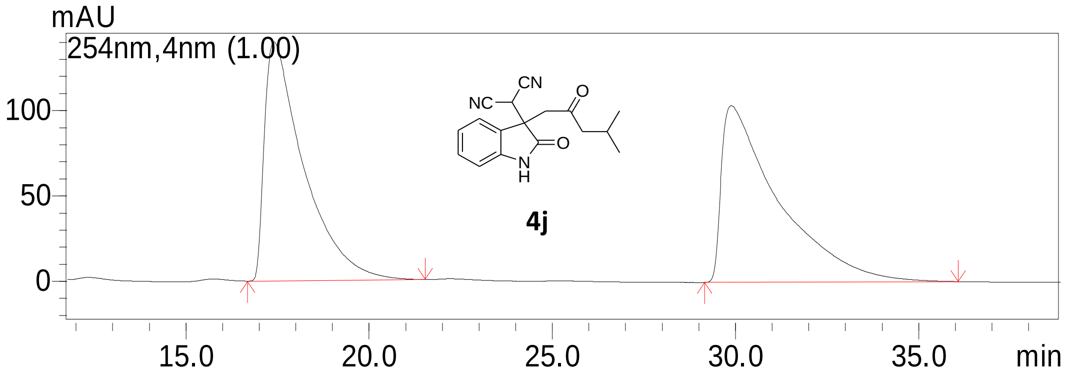

Peak	Ret. Time	Area%
1	17.401	48.2755
2	29.878	51.7245

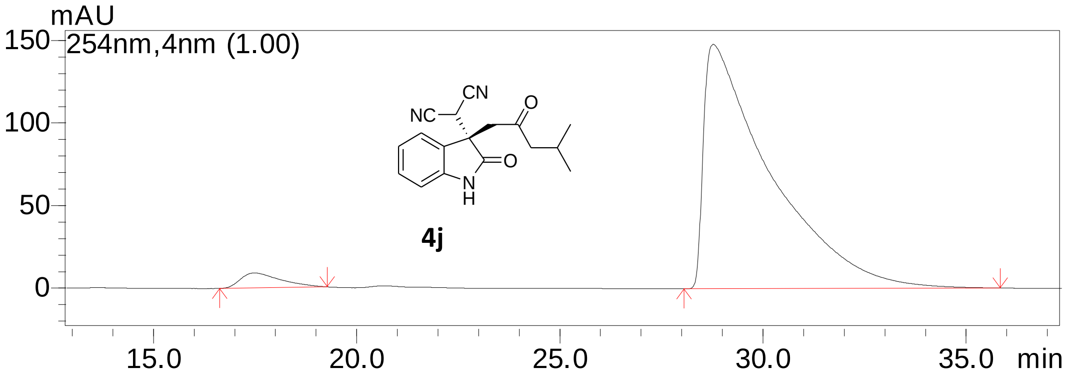

Peak	Ret. Time	Area%
1	17.477	3.5335
2	28.771	96.4665

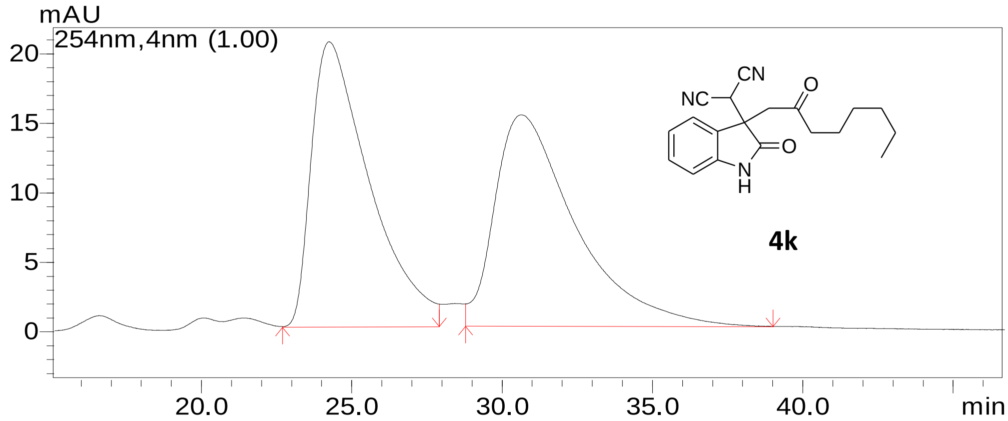

Peak	Ret. Time	Area%
1	24.239	49.1981
2	30.625	50.8019

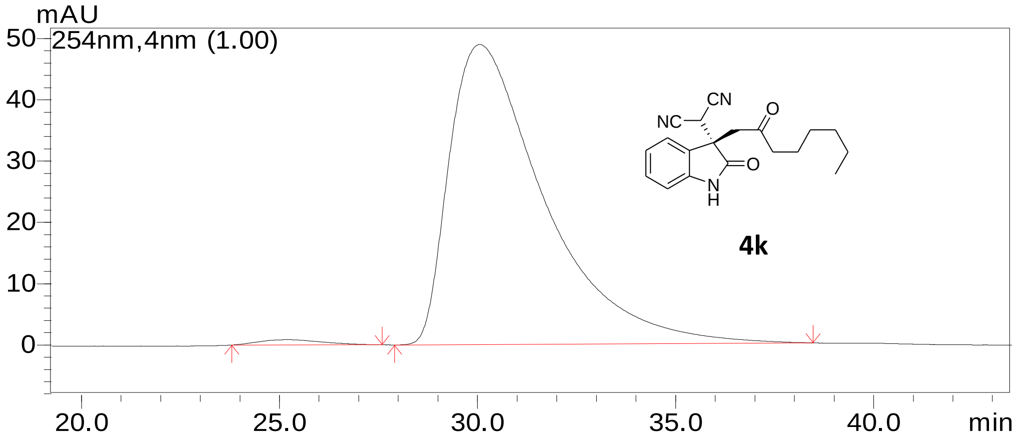

Peak	Ret. Time	Area%
1	25.286	1.1596
2	30.052	98.8404
